# Investigation of base excision repair gene variants in late-onset Alzheimer’s disease

**DOI:** 10.1371/journal.pone.0221362

**Published:** 2019-08-15

**Authors:** Tugce Ertuzun, Asli Semerci, Mehmet Emin Cakir, Aysegul Ekmekcioglu, Mehmet Oguz Gok, Daniela T. Soltys, Nadja C. de Souza-Pinto, Ugur Sezerman, Meltem Muftuoglu

**Affiliations:** 1 Department of Molecular Biology and Genetics; 2 Department of Medical Biotechnology, Acibadem Mehmet Ali Aydinlar University, Istanbul, Turkey; 3 Department of Neurology, Medeniyet University, Goztepe Training and Research Hospital, Istanbul, Turkey; 4 Departmento de Bioquímica, Instituto de Química, Universidade de São Paulo, São Paulo, Brazil; 5 Department of Biostatistics and Medical Informatics, Acibadem Mehmet Ali Aydinlar University, Istanbul, Turkey; Nathan S Kline Institute, UNITED STATES

## Abstract

Base excision repair (BER) defects and concomitant oxidative DNA damage accumulation play a role in the etiology and progression of late-onset Alzheimer’s disease (LOAD). However, it is not known whether genetic variant(s) of specific BER genes contribute to reduced BER activity in LOAD patients and whether they are associated with risk, development and/or progression of LOAD. Therefore, we performed targeted next generation sequencing for three BER genes, uracil glycosylase (*UNG*), endonuclease VIII-like DNA glycosylase 1 (*NEIL1*) and polymerase β (*POLβ*) including promoter, exonic and intronic regions in peripheral blood samples and *postmortem* brain tissues (temporal cortex, TC and cerebellum, CE) from LOAD patients, high-pathology control and cognitively normal age-matched controls. In addition, the known LOAD risk factor, *APOE* was included in this study to test whether any *BER* gene variants associate with *APOE* variants, particularly *APOE* ε4. We show that *UNG* carry five significant variants (rs1610925, rs2268406, rs80001089, rs1018782 and rs1018783) in blood samples of Turkish LOAD patients compared to age-matched controls and one of them (*UNG* rs80001089) is also significant in TC from Brazilian LOAD patients (p<0.05). The significant variants present only in CE and TC from LOAD are *UNG* rs2569987 and *POLβ* rs1012381950, respectively. There is also significant epistatic relationship (p = 0.0410) between *UNG* rs80001089 and *NEIL1* rs7182283 in TC from LOAD subjects. Our results suggest that significant BER gene variants may be associated with the risk of LOAD in non-*APOE* ε4 carriers. On the other hand, there are no significant *UNG*, *NEIL1* and *POLβ* variants that could affect their protein level and function, suggesting that there may be other factors such as post-transcriptional or–translational modifications responsible for the reduced activities and protein levels of these genes in LOAD pathogenesis. Further studies with increased sample size are needed to confirm the relationship between BER variants and LOAD risk.

## Introduction

Alzheimer`s disease (AD) is the most common cause of dementia in the aging population. AD is a progressive neurodegenerative disorder characterized by cognitive impairment, synaptic dysfunction, and pathological accumulation of extracellular amyloid-β (Aβ) plaques and intracellular neurofibrillary tangles (hyperphosphorylated tau proteins) [1]. The sporadic late-onset form of AD (LOAD) accounts for about 90% of AD cases (> 65 years). Although, the etiology and pathogenesis of LOAD are not fully understood, multiple environmental and epigenetic risk factors play a role in the development of the disease. Among LOAD susceptibility genes, the ε4 allele of the Apolipoprotein E gene (*APOE* ε4) is accepted as the strongest genetic risk factor. It has also been suggested that the presence of *APOE* ε4 may increase the rate of conversion from mild-cognitive impairment (MCI) to LOAD, and the disease progression. However, not all LOAD patients (up to 50%) carry the *APOE* ε4 allele and not all *APOE* ε4 carriers (up to 75%) develop LOAD [2–4]. Thus, uncovering new genetic risk factors for LOAD could shed new light into the understanding of the molecular mechanisms leading to the pathology.

Several studies have demonstrated that oxidative stress and concomitant oxidative DNA damage accumulation in nerve cells are also key factors in the onset and pathogenesis of LOAD [5–17]. The high metabolic rate of brain cells leads to increased production of the intracellular reactive oxygen species (ROS) which causes oxidative DNA damage. For example, nuclear and mitochondrial oxidative DNA lesions, including 8-hydroxyguanine (8-OHGua), 8-hydroxyadenine, 5-hydroxycytosine, 2,6-diamino-5-formamidopyrimidine (FapyAde), 4,6-diamino-5-formamidopyrimidine (FapyGua) and 5-hydroxyuracil (5-OHU) are found to be statistically significantly higher in lymphocytes, leukocytes and/or various brain regions of LOAD patients [5–15]. Moreover, increased oxidative DNA damage in MCI patients, which is considered to be a transition condition between normal aging and dementia, suggests that DNA oxidation may constitute an early event in the progression of LOAD [9]. The accumulation of oxidative DNA lesions is, in part, due to a deficiency in base excision repair (BER) capacity in LOAD and MCI patients [5,13,18]. BER is a major protective repair pathway for oxidative DNA lesions generated by endogenous sources, particularly ROS. BER mechanism is initiated by several different lesion-specific DNA glycosylases, such as uracil DNA glycosylase (UNG) and endonuclease VIII-like DNA glycosylase 1 (NEIL1) that recognize and remove oxidatively-induced damaged bases. Then, AP endonuclease 1 (APE1) processes abasic sites and generates a single nucleotide gap in the DNA. DNA polymerase β (POLβ) processes the ends and fills the gap and DNA ligase seals the nick to complete BER process [19,20].

The biochemical, cellular, molecular and behavioral studies performed with post-mortem brain tissues and peripheral blood samples of LOAD patients, Alzheimer mouse models and cell lines have revealed a strong correlation between BER deficiency and LOAD pathogenesis [5,13,18–30]. Several studies have demonstrated that the expression and activity of BER proteins are altered in LOAD progression [5,19,26,29]. UNG is a monofunctional DNA glycosylase involved in the first step of both nuclear and mitochondrial BER pathways. The *UNG* gene encodes both nuclear (*UNG2*) and mitochondrial (*UNG1*) isoforms of *UNG*, generated by alternative splicing [31,32]. It has been shown that LOAD and MCI brain tissues have decreased UNG activity and protein levels compared with normal brain tissues [5]. Recently, Soltys *et al*. have demonstrated that the activity of nuclear UNG was decreased in both cerebellum and temporal cortex of AD subjects whereas mitochondrial UNG activity was decreased only in temporal cortex [29]. The lack of UNG protein due to *UNG* gene silencing in rat hippocampal neurons caused neuronal death by inducing neuronal apoptosis, suggesting that this protein plays a crucial role in the neuronal development [21]. UNG excises uracil in DNA which accumulates due to spontaneous deamination of cytosine or dUTP misincorporation during replication. Unrepaired uracil lesions yield a mutagenic U:G or U:A mismatches. The accumulation of uracil due to a decrease in UNG activity and protein levels in nerve cells renders neurons more susceptible to Aβ-precursor protein toxicity and induces neuronal apoptosis [5,19,22,23]. NEIL1 DNA glycosylase is a bifunctional enzyme that has both glycosylase and AP endonuclease activities and excises FapyAde, FapyGua and 5-OHU base lesions. LOAD brain tissue exhibits a statistically significant decrease in NEIL1 protein levels and activity [26]. In addition, *NEIL1* gene expression levels were found decreased in lymphocytes from LOAD patients, which were not due to the methylation status of *NEIL1* gene promoter [33]. NEIL1 knockout mice studies have demonstrated that NEIL1 plays a crucial role in the prevention of short- and long-memory loss and cognitive decline [25]. Another key enzyme of the BER pathway is POLβ. In the 3xTg AD/Polβ^+/-^ mouse, POLβ depletion exacerbated neurodegeneration and AD phenotypes, including impaired memory retention, hippocampal synaptic plasticity and olfaction [27,28,30]. POLβ protein levels and single nucleotide gap filling activity were found to be statistically significantly reduced in brains from LOAD and MCI patients [5,24]. Weismann *et al*. showed that the defective BER capacity was due to deficiencies in UNG and POLβ activities in LOAD and MCI patients. It has been suggested that defective BER may play an important role in the progression of AD [5]. Lillines *et al*. demonstrated increased expression and protein levels of POLβ in the AD cerebellum compared to other brain regions and suggested that the high POLβ level of may correlate with late AD pathology [34]. Since BER deficiencies due to decrease in the activities and protein levels of UNG, NEIL1, and POLβ associated with LOAD pathogenesis, we have analyzed the impact of the variants of these three BER genes on the LOAD risk.

Genetic variant(s) of key BER genes responsible for the reduced BER activity in LOAD patients and LOAD development has not been thoroughly investigated yet. In recent years, functional variants and polymorphisms in BER genes that have been associated with increased risk for various types of cancer were analyzed in LOAD risk factor screening studies [35–46]. However, not all BER genes have been screened for their association with reduced BER capacity in LOAD patients and with LOAD development using targeted next generation sequencing (NGS) technology. Several studies have demonstrated no association between predominant variant of 8-oxoguanine DNA glycosylase (*OGG1*) gene, Ser326Cys, and LOAD risk [36,37,39,43]. Another mutations of *OGG1*, A53T, A288V and C796del, that cause a decrease in OGG1 activity have been identified in brain tissues of LOAD patients, but not in control tissues [41,42]. Since one patient has *OGG1* A53T, one patient has A288V and two patients have C796del out of 14 LOAD patients, large cohort studies are required for the association of these variants with LOAD risk [42]. No statistically significant association between the LOAD risk and several different BER gene variants has been identified, including *OGG1* Arg46Gln [37], *MUTYH* c.972G/C [35], *NEIL1* c.-283C/G [36], *APE1* (c.-468T/G and c.444T/G) [36,43], *FEN1* c.-441C/A [36], *LIG3* c.-50C/T [36] and *XRCC1* Arg280His, Arg399Gln and Arg194Trp [40,43,44]. However, Kwiatkowski et al. screened 110 patients and 120 healthy controls and found that G/A genotype of *XRCC1* rs25487 (Arg399Gln) increases the LOAD risk, but A/A genotype decreases the risk [35]. Lillenes et al. demonstrated the association of *APE1* c.444T/G with cognitive impairment independent of AD pathology [45]. Although there are no statistically significant differences in allele and genotype frequencies for *PARP1* rs1805404 (Asp81Asp) and rs1136410 (Val762Ala) between LOAD patients and control groups, two haplotypes (Ht3-TT and Ht4-CC) are associated with an increased risk of LOAD whereas a haplotype (Ht1-TC) showed a protective effect [46]. Kwiatkowski et. al showed that T/C genotype of *PARP1* Val762Ala is associated with LOAD risk but T/T variant reduced the risk. There is a relation between the genotypes of A/C and C/C in the *LIG3* c.83A>C and the A/A genotype of the *LIG1* c.-7C>T variant and LOAD risk [35].

In order to better understand the role of BER in LOAD, and to find out BER gene variants responsible for the reduced BER activity in LOAD patients, we evaluated the genetic variant(s) of three key BER genes, *UNG*, *NEIL1* and *POLβ*. For that, we performed targeted NGS for *UNG*, *NEIL1* and *POLβ* including promoter, exonic and intronic regions in peripheral blood samples from LOAD patients and cognitively age-matched normal controls as well as in postmortem brain tissues (temporal cortex and cerebellum) from LOAD patients, high-pathology control and cognitively normal controls. Furthermore, the known LOAD risk factor, *APOE* was also included in this study to see whether any of three BER gene variants associate with *APOE* variants, particularly *APOE* ε4, and whether this association contributes to LOAD risk. The present study also identified the distribution of *UNG*, *NEIL1* and *POLβ* variants for the first time in Turkish LOAD patients and healthy subjects.

## Materials and methods

### Study population

The peripheral blood samples were collected from 198 LOAD patients (>65 years) and 98 age-matched cognitively normal controls without any AD family history, recruited at the department of Neurology, Medeniyet University Goztepe Training and Research Hospital, Istanbul, Turkey. DNA samples from postmortem brain tissues (temporal cortex (TC) and cerebellum (CE)) from 11 LOAD, 10 cognitively normal control and 11 high-pathology control (hpC; cognitively normal with high AD neuropathological changes) were obtained from Dr. Nadja Souza Pinto, University of São Paulo, Brazil (the Brazilian Aging Brain Study Group’s Brain Bank, University of São Paulo, School of Medicine), as described in [29]. Written informed consents were obtained from all subjects prior to participation in this study. The study was approved by the Ethics Committee of Acibadem Mehmet Ali Aydinlar University and Acibadem Health Institutions Medical Research. The clinical diagnosis of LOAD was made according to the Neurological and Communicative Disorders and Stroke-Alzheimer’s Disease and Related Disorders Association (NINCDS-ADRDA) criteria and the criteria of Diagnostic and Statistical Manual of Mental disorders, 4^th^ ed. (DSM-IV). Cognitively normal participants received the same assessment as the cases and were accepted non-demented.

### DNA isolation

Total DNA was isolated using DNAeasy Blood & Tissue kit (Qiagen, Germany) according to the manufacturer`s protocol. DNA quality and quantity were evaluated using NanoDrop 2000c Spectrophotometer (Thermo Fisher Scientific, USA) and Qubit dsDNA HS Assay Kit (Thermo Fisher Scientific, USA) according to the manufacturer’s protocol.

### Targeted next generation gene sequencing

The Ion Torrent Personal Genome Machine (PGM) sequencing platform was used for the targeted *POLβ*, *UNG*, *NEIL1* and *APOE* genes sequencing according to the Ion Torrent protocols. *POLβ*, *UNG*, *NEIL1* and *APOE* gene primers including promoter, exon and intron regions (GRCh37-hg19 human reference genome) were designed using Ion Ampliseq Designer software (https://www.ampliseq.com) (Table 1). The primer sequences for each gene are shown in S1 Table, and the uncovered primer regions are shown in the gene structure maps (S1 Fig). The designed primer panel contains 226 amplicons in total and it is divided into two primer (amplicon) pools (113 amplicons each). The length of amplicons is between 125–375 bp (mean 268 ± 67.3 bp), the total size of primer panel is 60,030 bp and average gene coverage of primer panel is 94.5 ± 4.7% (Table 1). The Ion Torrent PGM sequencing was performed with high coverage 500X.

**Table 1:** The information for the designed Ion PGM primers using Ion Ampliseq Designer software.

	*POLβ* Chr8	*UNG* Chr12	*NEIL1* Chr15	*APOE* Chr19	*APOE* Promoter
**Amplicon’s beginning position**	42,195,472	109,534,879	75,637,831	45,409,033	45,408,011
**Amplicon’s ending position**	42,229,331	109,548,796	75,647,592	45,412,655	45,409,011
**Targeted base pair**	33859 bp	13919 bp	9761 bp	1220 bp	1000 bp
**Covered base pair**	29916 bp	13190 bp	8922 bp	1217 bp	980 bp
**Amplicon count**	124	54	36	8	4
**Coverage percentage**	88.35%	94.76%	91.4%	99.75%	98%

Library preparation was performed using the Ion AmpliSeq Library Kit 2.0 (Thermo Fisher Scientific, USA) according to the manufacturer’s protocol with some modifications. Briefly, 20 ng DNA was amplified with 1X Ion AmpliSeq HiFi Mix for each 1X primer pool using the Verity Thermal Cycler (Applied Biosystems, USA). Then, the samples were digested and phosphorylated with FuPa Reagent prior to ligating barcode adapters. Barcoded libraries were purified using the Agencourt AMPure XP Reagent (Beckman Coulter, USA). Purified libraries were amplified and purified using Agencourt AMPure XP Reagent (Beckman Coulter, USA). Amplified library concentrations were quantified and equalized to 100 pM using the Qubit dsDNA HS Assay Kit (Thermo Fisher Scientific, USA) according to manufacturer’s protocol. Template preparation was completed using the Ion PGM HiQ OT2 Kit (Thermo Fisher Scientific, USA) and Ion One Touch 2 Instrument (Thermo Fisher Scientific, USA) according to manufacturer’s protocol. Briefly, equalized libraries were mixed in equal volume and library mix was diluted into 8 pM. Diluted library was mixed with amplification solution containing Ion Sphere Particles (ISPs) and emulsion PCR was performed. Template positive ISPs were enriched using Ion OneTouch ES (Thermo Fisher Scientific, USA). Enriched template positive ISPs were sequenced using the Ion PGM HiQ Sequencing Kit (Thermo Fisher Scientific, USA) with Ion 318 Chip (Thermo Fisher Scientific, USA) in Ion Torrent PGM System (Thermo Fisher Scientific, USA) according to manufacturer’s protocol. Briefly, sequencing primer was annealed and sequencing polymerase was bound to template positive ISPs prior to loading onto Ion 318 Chip. After loading, PGM system was initialized.

### Bioinformatics and statistical analyses

Bioinformatics analysis of the raw data was performed using Torrent Suite Software v5.0.4 plugins (Thermo Fisher Scientific, USA). The results of Ion-PGM system were trimmed with the qualified standards of the system and aligned to GRCh37-hg19 human reference genome, and the VCF files were created using Variant caller plugin. The VCF files were analyzed using Ion Reporter Software (Thermo Fisher Scientific, USA) according to location, zygosity, position, type, and accession number of the variations. Then, the comparative analyses of case-control groups using the VCF files were performed by CLC Genomics Workbench (9.0.1., Qiagen, USA). The quality statistics of each dataset sequenced were determined using CLC Genomics Workbench (9.0.1., Qiagen, USA). Evaluation of statistically significantly important variations was done using Bonferroni corrected Fisher`s exact test p-value. Furthermore, the integrity of the sequenced amplicons was analyzed with Integrative Genomics Viewer (IGV) tool. The differences in variants between cases and controls were assessed by Pearson χ2 and Fisher`s exact tests. The χ2 test for Hardy-Weinberg equilibrium (HWE) was applied to each SNP among controls. For each SNP, we calculated odds ratio (OR) with 95% confidence interval (CI). We tested three different genetic models including dominant model, recessive model, and additive model [47]. The statistical power of the significant gene variations was calculated using G*Power software version 3.1.9.4 (Institute for experimental psychology in Dusseldorf, Germany). Linkage Disequilibrium (LD) and haplotype analysis of the identified SNPs were performed using Haploview 4.2 (Broad Institute of MIT and Harvard, Cambridge, MA, USA).

### Sanger sequencing

The potential variants identified by NGS were confirmed by Sanger sequencing. Sanger sequencing was performed using standard protocols. The Sanger primers are presented in S2 Table. Briefly, PCR products were purified using ExoSAP-IT PCR Product Cleanup Reagent (Applied Biosystems, USA) and then Big-Dye Terminator v3.1 Cycle-Sequencing Kit (Thermo Fisher Scientific, USA) was used according to manufacturer’s protocol. Purification of cycle sequencing PCR products was performed using Big-Dye XTerminator Purification Kit (Thermo Fisher Scientific, USA) according to manufacturer’s protocol. Sanger sequencing was performed using Applied Biosystems 3500DxGenetic Analyzer (Thermo Fisher, USA).

## Results

We performed targeted NGS for *UNG*, *NEIL1*, *POLβ* and *APOE* genes covering promoter, exonic and intronic regions on peripheral blood samples of 198 LOAD and 98 cognitively normal age-matched controls. The demographic and clinical characteristics of the participants are shown in Table 2. In addition, we performed targeted NGS of postmortem brain tissues from LOAD (10 TC and 11 CE), cognitively normal controls (9 TC and 10 CE) and hpC subjects (8 TC and 11 CE). The demographic, clinical and pathological characteristics of the postmortem brain tissues and their BER activities were reported in Soltys *et al*. 2019 [29].

**Table 2:** Characteristics of the study population.

Characteristics	LOAD, n = 198	Control, n = 98
**Age, mean ± SD**	79.85±7.83 (range: 65–97)	74.04±7.62 (range: 65–96)
**Female/Male**	118/80	57/41
**MMSE score**		
>20, mild (n)	21.38±1.77 (51)	
10–19, moderate (n)	15.13±2.56 (74)	Normal
<10, severe (n)	6.71±2.41 (73)	
**CDR score**	**n**	**n**
0, normal	0	98
1, mild	49	0
2, moderate	64	0
3, severe	85	**0**

MMSE, mini-mental state examination; CDR, clinical dementia rating scale; SD, standard deviation.

**Fig 1:**
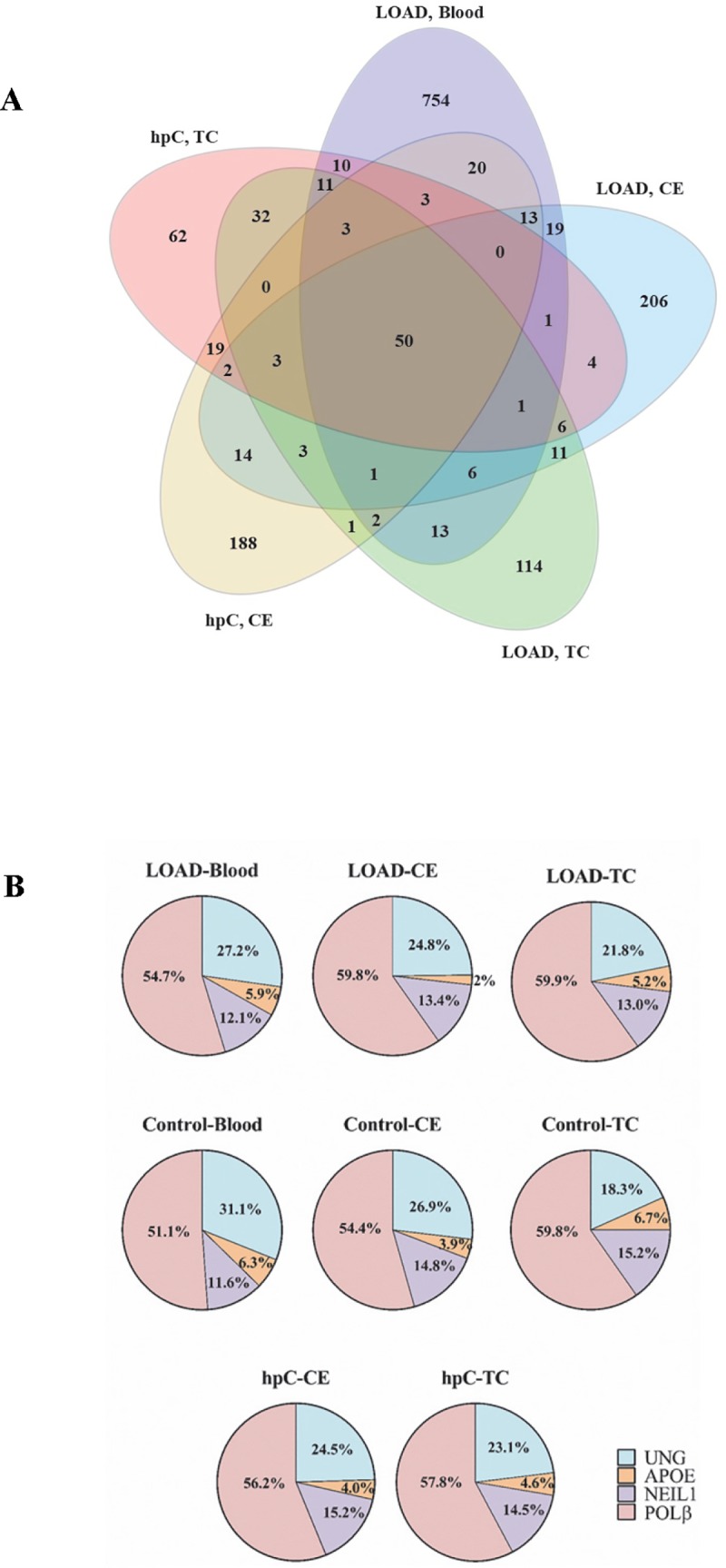
The distribution of genetic variants identified from NGS analysis in each sample. (A) The venn diagram showing the number of genetic variants in LOAD patients and hpC specific to or shared between the blood, TC and CE. (B) Pie charts showing the percent distribution of *UNG*, *NEIL1*, *POLβ* and *APOE* gene variants in each sample. LOAD, late-onset Alzheimer`s disease; CE, cerebellum; TC, temporal cortex; hpC, high-pathology control.

To assess the quality of the libraries sequenced, the basic quality statistics for Ion Torrent datasets were determined using CLC genomics workbench software. The quality distribution showed that more than 95% of the reads (total reads: 39,728,454) had average PHRED quality scores (Q score) over 20, with no ambiguous bases. The number of genetic variants identified from NGS analysis was as follows: 907 in LOAD and 544 in control blood samples; 403 in CE and 307 in TC of LOAD; 332 in CE and 282 in TC of hpC; 331 in CE and 328 in TC of cognitively controls (S3 Table). These variants were classified according to their distribution among tissues (Fig 1A). Among LOAD subjects, 81 variants were identical in CE and TC, 87 were identical in the TC and blood, and 91 were identical in CE and blood (Fig 1A). Fig 1B shows the percent distribution of *UNG*, *NEIL1*, *POLβ*, and *APOE* gene variants in each sample group. *POLβ* has the highest variant percentage, followed by *UNG*, *NEIL1* and *APOE* in each group (Fig 1B).

**Fig 2:**
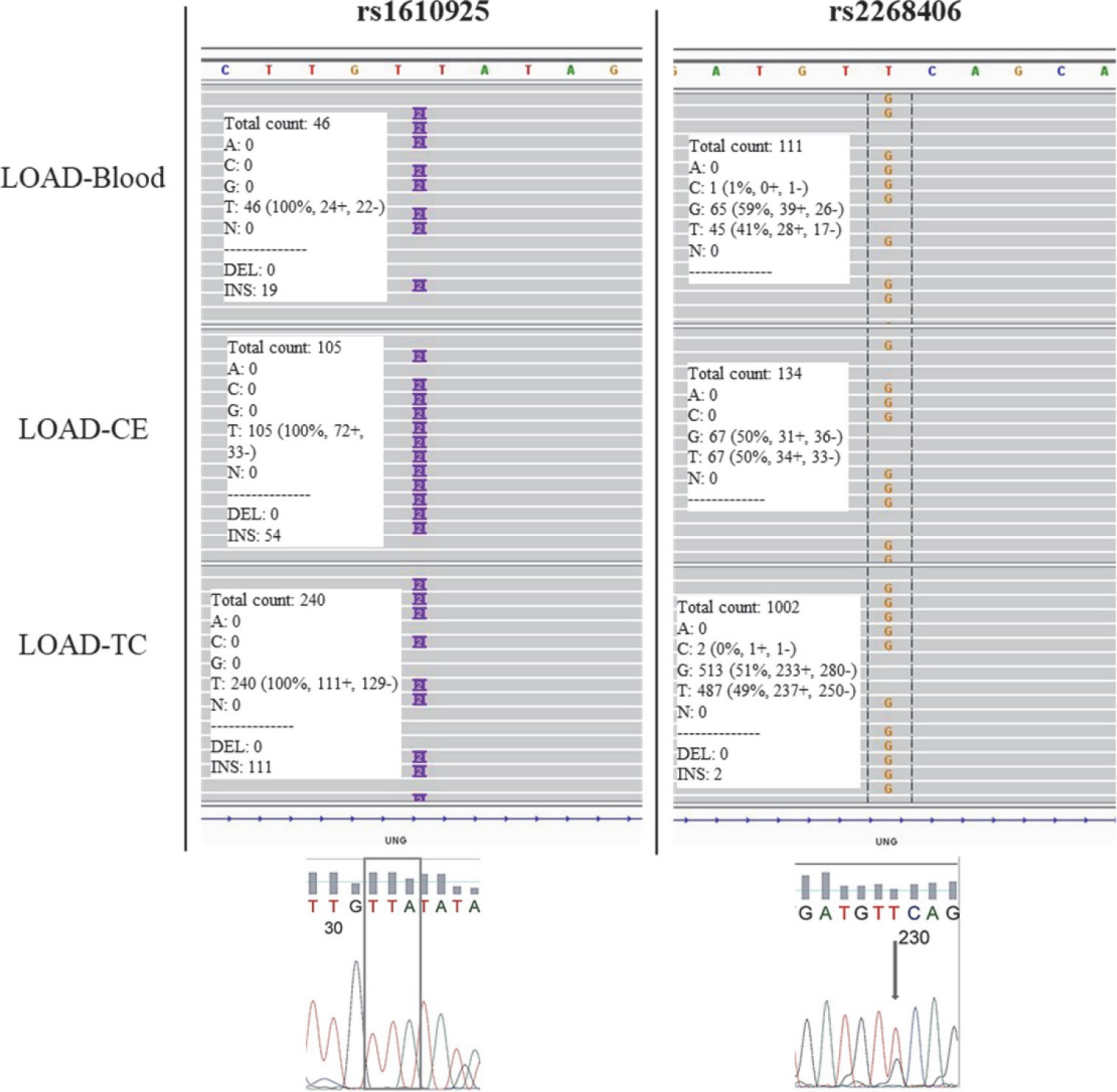
Identification and validation of genetic variants in *UNG* gene in LOAD patient’s blood, CE and TC samples. Targeted NGS results with corresponding Sanger sequencing validation of heterozygous variants *UNG* rs1610925 and rs2268406 in the blood of LOAD patients. NGS data are presented using the Integrative Genomics Viewer (IGV) software. Arrows and boxes indicate the position of the variant in the Sanger sequencing chromatograms. CE, cerebellum; TC, temporal cortex.

The statistical significance of the genetic variants associated with LOAD was evaluated for each SNP by p values of Fisher`s exact test. The genetic variants found in patients’ blood but not in more than 10% of controls were validated by Sanger sequencing. Five percent of the variants, mostly insertions/deletions (INDELs) and SNPs located in the repeated regions were not confirmed by Sanger sequencing. Fig 2 shows the IGV presentations and Sanger sequencing validation chromatograms of NGS results for *UNG* variants rs1610925 and rs2268406 in blood, cerebellum and temporal cortex samples from LOAD patients. Because of genomic mosaicism, it is difficult to confirm somatic gene variants by Sanger sequencing. Somatic variants with a relevant number of reads with the reference allele and/or the alternative allele were accepted as positive somatic variant (Fig 2).

### Allele and genotype frequencies between peripheral blood samples of LOAD patients and age-matched cognitively normal controls

The allelic and genotypic frequencies of *UNG*, *POLβ*, *NEIL1* and *APOE* in peripheral blood from LOAD patients and controls are presented in Tables 3 and 4. The gene variants that showed statistically significant deviation from the Hardy-Weinberg equilibrium (p<0.01) were excluded from further analysis. The allelic and genotypic frequencies of five *UNG* variants including an insertion rs1610925, and four SNPs, rs2268406, rs80001089, rs1018782 and rs1018783, were statistically significantly different (p<0.05) between LOAD and control groups in Turkish population (Table 3). The power analysis of statistically significant *UNG* and *APOE* variants showed that all statistically significant variants’ powers were between 81.8%-92.3%. *UNG* rs1610925 and *APOE* rs769449 SNPs had 85.6% power; *UNG* rs80001089 and rs1018783 SNP’s power was 92.3%; *UNG* rs2268406, and rs1018782 and *APOE* rs429358 SNP’s powers were 90.1%, 91.3% and 81.8%, respectively. The statistically significant SNPs of *UNG* were fitted into three different genetic models and all of them fit better to dominant and additive models (Table 5). Minor allele frequency (MAF) of statistically significant variants of our population were correlated with MAFs reported in 1000 Genome Project Phase 3 [48] (S4 Table). There was no statistically significant difference between the allelic and genotypic frequencies of *NEIL1* or *POLβ* variants between LOAD and control groups (Table 3). However, *POLβ* had three intronic variants, rs3136806 SNP (p = 0.0683), rs35609234 INDEL (p = 0.0706) and rs11990332 SNP (p = 0.0850), worth noticing. In addition, we identified two *NEIL1* noncoding transcript exon variants (mir631), rs10653888 INDEL and rs767369942 SNP, but they were not statistically significant (Table 3). Statistical analysis using three genetic models for *NEIL1* or *POLβ* variants showed no statistically significant difference between the LOAD patients and controls.

**Table 3:** Allele and genotype frequencies of *UNG*, *POLβ*, *NEIL1* and *APOE* in peripheral blood samples of LOAD patients and age-matched cognitively normal controls.

	Allele Frequency					Genotype Frequency						
	Allele	LOAD	CTRL	OR (95% CI)	Fisher`s p-value	Genotype	LOAD	CTRL	OR (95% CI)	Fisher`s p-value	Pearson χ2	P value
***UNG*, chr12**												
**rs1610925**	**-**	**0.803**	**0.913**	**2.58 (1.48–4.50)**	**0.0005**	**-/-**	**0.662**	**0.827**			**8.772**	**0.0031**
	**TA**	**0.197**	**0.087**			**-/TA**	**0.283**	**0.173**	**2.04 (1.11–3.75)**	**0.0224**		
						**TA/TA**	**0.055**	**0.000**	**-**	**0.0082**		
						**-/TA+TA/TA**	**0.338**	**0.173**	**2.44 (1.34–4.44)**	**0.0038**		
**rs2268406**	**T**	**0.836**	**0.918**	**2.21 (1.24–3.93)**	**0.0052**	**T/T**	**0.692**	**0.837**			**7.143**	**0.0075**
	**G**	**0.164**	**0.082**			**T/G**	**0.288**	**0.163**	**2.13 (1.15–3.96)**	**0.0153**		
						**G/G**	**0.020**	**0.000**	**-**	**0.2993**		
						**T/G+G/G**	**0.308**	**0.163**	**2.28 (1.23–4.22)**	**0.0075**		
**rs80001089**	**T**	**0.891**	**0.954**	**2.53 (1.21–5.30)**	**0.0129**	**T/T**	**0.788**	**0.908**			**6.651**	**0.0099**
	**G**	**0.109**	**0.046**			**T/G**	**0.207**	**0.092**	**2.60 (1.21–5.60)**	**0.0131**		
						**G/G**	**0.005**	**0.000**	**-**	**1.0000**		
						**T/G+G/G**	**0.212**	**0.092**	**2.66 (1.24–5.72)**	**0.0091**		
**rs1018782**	**A**	**0.848**	**0.918**	**2.01 (1.12–3.59)**	**0.0184**	**A/A**	**0.717**	**0.837**			**5.091**	**0.0241**
	**G**	**0.152**	**0.082**			**A/G**	**0.263**	**0.163**	**1.88 (1.01–3.50)**	**0.0563**		
						**G/G**	**0.020**	**0.000**	**-**	**0.2995**		
						**A/G+G/G**	**0.283**	**0.163**	**2.02 (1.09–3.75)**	**0.0304**		
**rs1018783**	**T**	**0.866**	**0.923**	**1.86 (1.02–3.40)**	**0.0406**	T/T	0.753	0.847			3.448	0.0633
	**A**	**0.134**	**0.077**			T/A	0.227	0.153	1.67 (0.88–3.18)	0.1271		
						A/A	0.020	0.000	-	0.3004		
						T/A+A/A	0.247	0.153	1.82 (0.96–3.44)	0.0723		
rs2430678	A	0.980	1.000	-	0.0575	**A/A**	**0.960**	**1.000**			**4.070**	**0.0437**
	G	0.020	0.000			**A/G**	**0.040**	**0.000**	**-**	**0.0558**		
						**G/G**	**0.000**	**0.000**	**-**	**1.0000**		
						**A/G+G/G**	**0.040**	**0.000**	**-**	**0.0558**		
***NEIL1*, chr15**												
rs10653888	-	0.967	0.990	3.29 (0.74–14.74)	0.1618	-/-	0.934	0.980			2.790	0.0949
	ACACACAC	0.033	0.010			-/ACACACAC	0.066	0.020	3.37 (0.75–15.25)	0.1563		
						ACACACAC/ ACACACAC	0.000	0.000	-	1.0000		
						-/ACACACAC + ACACACAC/ ACACACAC	0.066	0.020	3.37 (0.75–15.25)	0.1563		
rs5745916	G	0.942	0.969	1.95 (0.78–4.88)	0.1619	G/G	0.884	0.939			2.239	0.1346
	A	0.058	0.031			G/A	0.116	0.061	2.01 (0.79–5.12)	0.1511		
						A/A	0.000	0.000	-	1.0000		
						G/A+A/A	0.116	0.061	2.01 (0.79–5.12)	0.1511		
rs11634109	T	0.891	0.923	1.47 (0.80–2.72)	0.2422	T/T	0.788	0.847			1.471	0.2252
	C	0.109	0.077			T/C	0.207	0.153	1.45 (0.76–2.78)	0.2746		
						C/C	0.005	0.000	-	1.0000		
						T/C+C/C	0.212	0.153	1.49 (0.78–2.85)	0.2735		
rs767369942	G	0.980	0.995	4.02 (0.50–32.38)	0.2839	G/G	0.960	0.990			2.028	0.1544
	A	0.020	0.005			G/A	0.040	0.010	4.08 (0.50–33.13)	0.2801		
						A/A	0.000	0.000	-	1.0000		
						G/A+A/A	0.040	0.010	4.08 (0.50–33.13)	0.2801		
***POLβ*, chr8**												
rs3136806	T	0.912	0.954	2.01 (0.95–4.28)	0.0683	T/T	0.823	0.908			3.736	0.0532
	G	0.088	0.046			T/G	0.177	0.092	2.12 (0.98–4.62)	0.0575		
						G/G	0.000	0.000	-	1.0000		
						T/G+G/G	0.177	0.092	2.12 (0.98–4.62)	0.0575		
rs35609234	C	0.871	0.923	1.78 (0.97–3.26)	0.0706	C/C	0.763	0.857			3.579	0.0585
	-	0.129	0.077			C/-	0.217	0.133	1.84 (0.94–3.62)	0.0836		
						-/-	0.020	0.010	2.22 (0.24–20.23)	0.6585		
						C/-+-/-	0.237	0.143	1.87 (0.97–3.59)	0.0672		
rs11990332	A	0.881	0.929	1.75 (0.94–3.26)	0.0850	A/A	0.783	0.867			3.053	0.0806
	G	0.119	0.071			A/G	0.197	0.122	1.78 (0.89–3.58)	0.1403		
						G/G	0.020	0.011	2.19 (0.24–19.94)	0.6597		
						A/G+G/G	0.217	0.133	1.81 (0.92–3.56)	0.0852		
rs571459229	G	0.985	1.000	-	0.1853	G/G	0.970	1.000			3.031	0.0817
	T	0.015	0.000			G/T	0.030	0.000	-	0.1830		
						T/T	0.000	0.000	-	1.0000		
						G/T+T/T	0.030	0.000	-	0.1830		
rs3136744	A	0.957	0.974	1.71 (0.62–4.71)	0.3608	A/A	0.914	0.949			1.156	0.2822
	C	0.043	0.026			A/C	0.086	0.051	1.75 (0.62–4.88)	0.3513		
						C/C	0.000	0.000	-	1.0000		
						A/C+C/C	0.086	0.051	1.75 (0.62–4.88)	0.3513		
***APOE*, chr19**												
**rs769449**	**G**	**0.866**	**0.974**	**5.90 (2.32–15.02)**	**0.0001**	**G/G**	**0.747**	**0.949**			**17.594**	**0.0001**
	**A**	**0.134**	**0.026**			**G/A**	**0.237**	**0.051**	**5.91 (2.27–15.39)**	**0.0001**		
						**A/A**	**0.015**	**0.000**	**-**	**0.2895**		
						**G/A+A/A**	**0.253**	**0.051**	**6.28 (2.42–16.33)**	**0.0001**		
**rs429358**	**T**	**0.811**	**0.939**	**3.58 (1.89–6.76)**	**0.0001**	**T/T**	**0.652**	**0.878**			**16.851**	**0.0001**
	**C**	**0.189**	**0.061**			**T/C**	**0.318**	**0.122**	**3.50 (1.78–6.87)**	**0.0001**		
						**C/C**	**0.030**	**0.000**	**-**	**0.0839**		
						**T/C+C/C**	**0.348**	**0.133**	**3.83 (1.96–7.50)**	**0.0001**		

**Table 4:** Allele and genotype frequencies of *APOE* ε2, ε3 and ε4 in peripheral blood samples of LOAD patients and age-matched cognitively normal controls.

rs429358-rs7412					
		LOAD Frequency	Control Frequency	OR (95% CI)	Fisher’s p-value
**Allele**					
ε2	T-T	0.0354	0.0408	1.00 (0.41–2.44)	1.0000
ε3	T-C	0.7753	0.8980	1 (REF)	REF
ε4	C-C	0.1894	0.0612	3.58 (1.89–6.77)	0.0001
**Genotype**					
ε2/ε2	TT-TT	0.000	0.000	-	1.0000
ε2/ε3	TT-TC	0.056	0.061	1.24 (0.44–3.50)	0.7993
ε2/ε4	TC-TC	0.015	0.020	1.02 (0.17–6.22)	1.0000
ε3/ε3	TT-CC	0.601	0.816	1 (REF)	REF
ε3/ε4	TC-CC	0.298	0.102	4.07 (1.97–8.42)	0.0001
ε4/ε4	CC-CC	0.030	0.000	-	0.0834

**Table 5:** Analysis of gene variants in blood samples based on genetic models.

	Additive Model		Dominant Model		Recessive Model	
	OR (95% CI)	Fisher’s p-value	OR (95% CI)	Fisher’s p-value	OR (95% CI)	Fisher’s p-value
**rs1610925**	2.58 (1.48–4.50)	0.0005	2.44 (1.34–4.44)	0.0038	-	0.0183
**rs2268406**	2.76 (1.56–4.87)	0.0002	2.62 (1.42–4.83)	0.0015	-	0.0183
**rs80001089**	2.53 (1.21–5.30)	0.0129	2.66 (1.24–5.72)	0.0091	-	1.0000
**rs1018782**	2.19 (1.23–3.90)	0.0184	2.02 (1.08–3.75)	0.0304	-	0.3058
**rs1018783**	1.86 (1.02–3.40)	0.0406	1.82 (0.96–3.44)	0.0723	-	0.3058
**rs2430678**	-	0.0575	-	0.0558	-	1.0000

**Fig 3:**
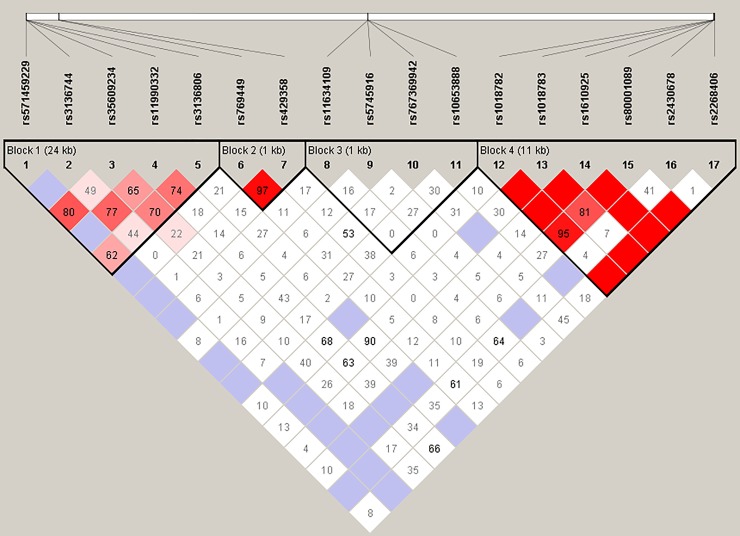
Linkage disequilibrium plot of all studied LOAD blood gene variants. D' (pairwise SNP correlation) values were represented in the boxes. The plot’s block 1, 2, 3 and 4 were defined according to the genes in the following order; *POLβ*, *APOE*, *NEIL1* and *UNG*.

Linkage disequilibrium (LD) results of the all studied LOAD-blood variants were shown in Fig 3 LD plot. D' (pairwise SNP correlation) values were represented on the plot and blocks were defined according to the genes. *UNG* gene variant pairs rs1018782-rs1018783, rs1018783-rs1610925, rs1610925-rs80001089, rs1018782-rs1610925, rs1610925-rs2430687, rs1018782-rs2268406, rs1018783-rs2268406, rs1610925-rs2268406 and rs80001089-rs2268406 were in complete LD. *UNG* gene variant pair rs1018782-rs80001089 and *APOE* gene variant pair rs769449-rs429358 were in strong LD with r^2^≥0.50 and D' approaching to 1. There was no strong LD between *NEIL1* and *POLβ* gene variant pairs. *POLβ* GAAGG, *APOE* AC and *UNG* GAAGAG haplotypes were found statistically significantly different (p<0.05) between LOAD and control groups in Turkish population. *POLβ* GACAT, *APOE* GT, *NEIL1* TGGA and *UNG* ATTTAT haplotypes were found statistically significantly higher (p<0.05) in control group suggesting a protective effect against LOAD (Table 6). In addition, to study the combinatorial effects of the variants, we carried out epistatis analysis. The epistatic relationships between the different *UNG* variants (rs80001089-rs1610925; rs1610925-rs2268406; rs80001089-rs2268406; rs80001089-rs1018782; rs2268406-rs1018782; rs1610925-rs1018782) and the *APOE* variants rs429358-rs769449 were found statistically significant (p<0.05) between each other, but not among them (S5 Table).

**Table 6:** Haplotype analysis of all studied LOAD blood gene variants.

**Gene**	SNP #	Haplotype	All (%)	Case (%)	Control (%)	OR (95% CI)	Fisher`s p-value	Chi Square	P Value
Block 1									
*POLβ*	1,2,3,4,5	GACAT	0.902	0.871	0.965	0.24 (0.11–0.54)	0.0001	13.188	0.0003
		GAAGG	0.021	0.032	0.000	-	0.0063	6.494	0.0108
Block 2									
*APOE*	6,7	GT	0.853	0.810	0.939	0.27 (0.15–0.52)	0.0001	17.529	0.00003
		AC	0.096	0.131	0.025	5.83 (2.29–14.86)	0.0001	17.101	0.00004
		GC	0.049	0.056	0.035	1.60 (0.67–3.82)	0.3194	1.168	0.2798
Block 3									
*NEIL1*	8,9,10,11	TGGA	0.837	0.813	0.886	0.57 (0.34–0.94)	0.0336	5.185	0.0228
		CGGA	0.089	0.097	0.073	0.49 (0.30–0.79)	0.2888	0.914	0.339
		TAGA	0.035	0.040	0.023	2.04 (0.67–6.19)	0.2354	1.222	0.269
		TGGC	0.012	0.013	0.010	1.25 (0.24–6.52)	1.0000	0.073	0.7866
Block 4									
*UNG*	12,13,14,15,16,17	ATTTAT	0.848	0.816	0.914	0.42 (0.24–0.73)	0.0015	9.959	0.0016
		GAAGAG	0.074	0.091	0.040	2.37 (1.08–5.21)	0.0205	4.909	0.0267
		GAATAG	0.040	0.043	0.035	1.22 (0.50–3.00)	0.8258	0.195	0.6585
		ATATGT	0.012	0.018	0.000	-	0.1018	2.031	0.1541
		GTAGAG	0.010	0.013	0.005	2.52 (0.29–21.71)	0.6691	0.758	0.3841

*APOE* variants, rs429358 (Cys130Arg) and rs769449 showed statistically significant association with LOAD (Table 3). *APOE* gene contains three major allelic variants (ε2, ε3, and ε4) encoding different isoforms (ApoE2, ApoE3, and ApoE4) that differ only in two SNPs (rs429358 and rs7412). They generate three homozygous (*ε*2/*ε*2, *ε*3/*ε*3 and *ε*4/*ε*4) and three heterozygous (*ε*2/*ε*3, *ε*2/*ε*4 and *ε*3/*ε*4) genotypes [2–4]. Allele and genotype frequencies of *APOE* ε2, ε3, and ε4 in LOAD patients and cognitively normal controls are shown in Table 4. The allele frequency of ε3 (0.7753 in LOAD and 0.8980 in control) and the genotype frequency of ε3/ε3 (0.601 in LOAD and 0.816 in control) were much higher than either that of ε2 or ε4 and ε2/ε3, ε2/ε4, ε3/ε4 or ε4/ε4. The *APOE* ε4 allele frequency and ε3/ε4 genotype frequency in LOAD patients was statistically significantly higher compared with that of the control group (p = 0.0001) (Table 4). The significance of homozygote ε4/ε4 genotype was p = 0.0834. Furthermore, we evaluated the effects of the interaction of statistically significant variants (Table 3) with *APOE* ε4 carriers or non-carriers in LOAD case-control status (Table 7). Individuals carrying the *APOE* genotype ε2/ε4 (3 LOAD and 2 controls) were excluded from this analysis for having both protective and risk alleles. *UNG* rs1610925, rs2268406, 80001089, rs1018782 and rs1018783 increase the risk of LOAD in Turkish population statistically significantly in non-*APOE* ε4 carriers, but not in *APOE* ε4 carriers. For example, the risk of LOAD is statistically significantly higher for *UNG* rs80001089 carriers in the absence of *APOE* ε4 (OR = 6.03, 95% CI = 2.04–17.84, p = 0.0002) compared to *APOE* ε4 carriers (OR = 3.58) or *UNG* rs80001089 carriers (OR = 2.53) alone. *APOE* rs769449 was found statistically significant in *APOE* ε4 carriers (Table 7).

**Table 7:** Effect of the interaction of *UNG* and *APOE* variants with *APOE* ε4 in blood samples of LOAD case-control status.

		LOAD	Control	OR (95% CI)	Fisher’s p-value
APOE ε4 (+)	rs769449 (+)	24%	3%	6.09 (1.42–26.17)	0.0131
	rs769449 (-)	9%	7%		
APOE ε4 (-)	rs769449 (+)	1%	0%	-	1.0000
	rs769449 (-)	66%	90%		
APOE ε4 (+)	rs1610925 (+)	11%	4%	0.70 (0.18–2.75)	0.7210
	rs1610925 (-)	23%	6%		
APOE ε4 (-)	rs1610925 (+)	24%	11%	4.00 (1.94–8.26)	0.0001
	rs1610925 (-)	42%	79%		
APOE ε4 (+)	rs2268406 (+)	10%	4%	0.67 (0.17–2.62)	0.7173
	rs2268406 (-)	23%	6%		
APOE ε4 (-)	rs2268406 (+)	24%	11%	3.83 (1.85–7.93)	0.0001
	rs2268406 (-)	43%	79%		
APOE ε4 (+)	rs80001089 (+)	6%	3%	0.53 (0.12–2.35)	0.4084
	rs80001089 (-)	27%	7%		
APOE ε4 (-)	rs80001089 (+)	15%	4%	6.03 (2.04–17.84)	0.0002
	rs80001089 (-)	52%	86%		
APOE ε4 (+)	rs1018782 (+)	8%	3%	0.70 (0.16–3.05)	0.6950
	rs1018782 (-)	26%	7%		
APOE ε4 (-)	rs1018782 (+)	21%	11%	3.11 (1.50–6.48)	0.0019
	rs1018782 (-)	46%	79%		
APOE ε4 (+)	rs1018783 (+)	6%	3%	0.53 (0.12–2.35)	0.4084
	rs1018783 (-)	27%	7%		
APOE ε4 (-)	rs1018783 (+)	19%	11%	2.78 (1.33–5.82)	0.0073
	rs1018783 (-)	48%	79%		
APOE ε4 (+)	rs2430678 (+)	2%	0%	-	1.0000
	rs2430678 (-)	31%	10%		
APOE ε4 (-)	rs2430678 (+)	3%	0%	-	0.1600
	rs2430678 (-)	64%	90%		
rs769449 (+)	APOE ε4 (+)	24%	3%	-	1.0000
	APOE ε4 (-)	1%	0%		
rs769449 (-)	APOE ε4 (+)	9%	7%	1.75 (0.70–4.38)	0.2817
	APOE ε4 (-)	66%	90%		
rs1610925 (+)	APOE ε4 (+)	11%	4%	1.20 (0.34–4.22)	1.0000
	APOE ε4 (-)	24%	11%		
rs1610925 (-)	APOE ε4 (+)	23%	6%	6.88 (2.78–17.02)	0.0001
	APOE ε4 (-)	42%	79%		
rs2268406 (+)	APOE ε4 (+)	10%	4%	1.20 (0.34–4.21)	1.0000
	APOE ε4 (-)	24%	11%		
rs2268406 (-)	APOE ε4 (+)	23%	6%	6.88 (2.78–17.02)	0.0001
	APOE ε4 (-)	43%	79%		
rs80001089 (+)	APOE ε4 (+)	6%	3%	0.55 (0.11–2.85)	0.6615
	APOE ε4 (-)	15%	4%		
rs80001089 (-)	APOE ε4 (+)	27%	7%	6.30 (2.72–14.58)	0.0001
	APOE ε4 (-)	52%	86%		
rs1018782 (+)	APOE ε4 (+)	8%	3%	1.38 (0.34–5.62)	0.7471
	APOE ε4 (-)	21%	11%		
rs1018782 (-)	APOE ε4 (+)	26%	7%	6.11 (2.62–14.26)	0.0001
	APOE ε4 (-)	46%	79%		
rs1018783 (+)	APOE ε4 (+)	6%	3%	1.19 (0.28–4.98)	1.0000
	APOE ε4 (-)	19%	11%		
rs1018783 (-)	APOE ε4 (+)	27%	7%	6.27 (2.70–14.58)	0.0001
	APOE ε4 (-)	48%	79%		
rs2430678 (+)	APOE ε4 (+)	2%	0%	-	1.0000
	APOE ε4 (-)	3%	0%		
rs2430678 (-)	APOE ε4 (+)	31%	10%	4.30 (2.09–8.83)	0.0001
	APOE ε4 (+)	64%	90%		

We analyzed the *UNG* gene expression levels in six LOAD patients who carry statistically significant *UNG* gene variants (two of them have rs1610925, rs2268406, rs1018782 and rs101878, and four of them have rs1610925, rs2268406, and rs80001089) and four cognitively normal age-matched controls who do not carry these *UNG* variants. There was no statistically significant difference in *UNG* gene expression among LOAD patients and controls in relation to the *UNG* genotype (p>0.05) (S2 Fig and S1 Text).

### Allele and genotype frequencies in postmortem brain tissues from LOAD, hpC and age-matched cognitively normal controls

We performed targeted NGS analysis of 10 TC and 11 CE of LOAD, 9 TC and 10 CE of cognitively normal controls, and 8 TC and 11 CE of hpC samples. The allele and genotype frequencies of CE and TE in LOAD, control and hpC subjects are presented in S6 and S7 Tables. The allele and genotype frequencies of *UNG* rs2569987 was found statistically significantly different between LOAD and control in cerebellum (p<0.05) (Table 8 and S6 Table). Because hpC individuals show neuropathological features of AD, but remained cognitively normal, for these analysis these individuals were included in the control group. The allele and genotype frequencies of *UNG* rs2569987 were also found statistically significantly different between LOAD-CE and control+hpC-CE (p<0.05), but not between hpC-CE and control-CE (Table 8 and S6 Table). Because of the low DNA quality of TC samples, we could not perform targeted NGS analysis for both brain regions for all samples. We compared the variant profile of CE and TC of each postmortem brain tissue for LOAD (10 samples for each region), cognitively normal controls (9 samples for each region) and hpC group (8 samples for each region) (Table 9). No statistically significant differences (p<0.05) were found between *UNG* and *NEIL1* allele and genotype frequencies when comparing LOAD-CE or–TC and control-CE or–TC (Table 9). The genotype frequency of *POLβ* rs1012381950 was found statistically significant difference (p = 0.0198) between LOAD-TC and control-TC whereas the allele frequency of this variant was not statistically significant (p = 0.0860) (Table 9). The allele and genotype frequencies of *APOE* rs405509 was found to be statistically significantly different (p = 0.006 and p = 0.009, respectively) between hpC-CE and cognitively normal control-CE (Table 9). *UNG* rs80001089 allele and genotype frequencies were found to be statistically significantly different (p = 0.0153 and p = 0.012, respectively) between TC of LOAD and TC of control group (hpC+control) (Table 9). On the other hand, p value for *UNG* rs80001089 was 0.1071 when comparing LOAD-TC and control-TC (Table 9).

**Table 8:** Allele and genotype frequencies of *UNG* rs2569987 in CE of LOAD patients, hpC and age-matched cognitively normal controls subjects (LOAD = 11, hpC = 11, Control = 10).

*UNG* rs2569987											
Allele frequency					Genotype frequency						
**Allele**	**LOAD**	**Control**	**OR (95% CI)**	**Fisher’s p-value**	**Genotype**	**LOAD**	**Control**	**OR (95% CI)**	**Fisher’s p-value**	**Pearson χ2**	**P value**
**T**	0.73	1.00	-	0.0216	T/T	0.55	1.00			5.966	0.0146
**C**	0.27	0.00			T/C	0.36	0.00	-	0.0867		
					C/C	0.09	0.00	-	0.4118		
** **					T/C+C/C	0.45	0.00	-	0.0351		
	**LOAD**	**hpC + Control**				**LOAD**	**hpC+ Control**				
**T**	0.73	0.95	7.50 (1.37–41.14)	0.0162	T/T	0.55	0.90			5.453	0.0195
**C**	0.27	0.05			T/C	0.36	0.10	6.33 (0.92–43.62)	0.0674		
					C/C	0.09	0.00	-	0.2692		
					T/C+C/C	0.45	0.10	7.92 (1.21–51.84)	0.0318		

**Table 9:** Comparison of allele and genotype frequencies of *UNG*, *POLβ*, *NEIL1* and *APOE* in CE and TC samples of same LOAD patients, hpC and age-matched cognitively normal controls (LOAD = 10, hpC = 8, Control = 9).

	**LOAD vs Control**												
		**Allele frequency**					**Genotype frequency**						
		**Allele**	**LOAD**	**Control**	**OR (95% CI)**	**Fisher’s p-value**	**Genotype**	**LOAD**	**Control**	**OR (95% CI)**	**Fisher’s p-value**	**Pearson χ2**	**P value**
***UNG*, chr12**													
**CE**	rs2569987	T	0.80	1.00	-	0.1071	T/T	0.60	1.00			4.560	0.0327
		C	0.20	0.00			T/C	0.40	0.00	-	0.0867		
							C/C	0.00	0.00	-	1.0000		
							T/C+C/C	0.40	0.00	-	0.0867		
**TC**	rs80001089	T	0.80	1.00	-	0.1071	T/T	0.60	1.00			4.560	0.0327
		G	0.20	0.00			T/G	0.40	0.00	-	0.0867		
							G/G	0.00	0.00	-	1.0000		
							T/G+G/G	0.40	0.00	-	0.0867		
	rs2569987	T	0.80	1.00	-	0.1071	T/T	0.60	1.00			4.560	0.0327
		C	0.20	0.00			T/C	0.40	0.00	-	0.0867		
							C/C	0.00	0.00	-	1.0000		
							T/C+C/C	0.40	0.00	-	0.0867		
***NEIL1*, chr15**													
	rs7182283	G	0.40	0.67	3.00 (0.80–11.31)	0.1192	G/G	0.20	0.56			2.574	0.1087
		T	0.60	0.33			G/T	0.40	0.22	5.00 (0.47–52.96)	0.2861		
**CE**							T/T	0.40	0.22	5.00 (0.47–52.96)	0.2861		
							G/T+T/T	0.80	0.44	5.00 (0.66–38.15)	0.1698		
	rs7182283	G	0.40	0.67	3.00 (0.80–11.31)	0.1192	G/G	0.20	0.56			2.574	0.1087
		T	0.60	0.33			G/T	0.40	0.22	5.00 (0.47–52.96)	0.2861		
**TC**							T/T	0.40	0.22	5.00 (0.47–52.96)	0.2861		
							G/T+T/T	0.80	0.44	5.00 (0.66–38.15)	0.1698		
***POLβ*, chr8**													
**TC**	**rs1012381950**	**T**	**0.55**	**0.83**	**4.09 (0.89–18.72)**	**0.0860**	**T/T**	**0.10**	**0.67**			**6.537**	**0.0106**
		**C**	**0.45**	**0.17**			**T/C**	**0.90**	**0.33**	**18.00 (1.50–216.63)**	**0.0198**		
							**C/C**	**0.00**	**0.00**	**-**	**1.000**		
							**T/C+C/C**	**0.90**	**0.33**	**18.00 (1.50–216.63)**	**0.0198**		
**hpC vs Control**													
		**Allele frequency**					**Genotype frequency**						
		**Allele**	**hpC**	**Control**	**OR (95% CI)**	**Fisher’s p-value**	**Genotype**	**hpC**	**Control**	**OR (95% CI)**	**Fisher’s p-value**	**Pearson χ2**	**P value**
***APOE*, chr19**													
**CE**	**rs405509**	**T**	**0.63**	**1.00**	**-**	**0.0060**	**T/T**	**0.38**	**1.00**			**7.969**	**0.0048**
		**G**	**0.37**	**0.00**		** **	**T/G**	**0.50**	**0.00**	**-**	**0.0192**		** **
						** **	**G/G**	**0.13**	**0.00**	**-**	**0.3077**		** **
		** **	** **	** **	** **	** **	**T/G+G/G**	**0.63**	**0.00**	**-**	**0.0090**	** **	** **
**LOAD vs hpC+Control**													
		**Allele frequency**					**Genotype frequency**						
		**Allele**	**LOAD**	**hpC+** **Control**	**OR (95% CI)**	**Fisher’s p-value**	**Genotype**	**LOAD**	**hpC + Control**	**OR (95% CI)**	**Fisher’s p-value**	**Pearson χ2**	**P value**
***UNG*, chr12**													
	rs80001089	T	0.90	1.00	-	0.1328	T/T	0.80	1.00			3.672	0.0553
		G	0.10	0.00			T/G	0.20	0.00	-	0.1282		
							G/G	0.00	0.00	-	1.0000		
**CE**							T/G+G/G	0.20	0.00	-	0.1282		
	rs2569987	T	0.80	0.94	4.00 (0.66–24.21)	0.1792	T/T	0.60	0.88			2.904	0.0883
		C	0.20	0.06			T/C	0.40	0.12	5.00 (0.72–34.94)	0.1535		
							C/C	0.00	0.00	-	1.0000		
							T/C+C/C	0.40	0.12	5.00 (0.72–34.94)	0.1535		
	rs2268406	T	0.80	0.94	4.00 (0.66–24.21)	0.1792	T/T	0.60	0.88			2.904	0.0883
		G	0.20	0.06			T/G	0.40	0.12	5.00 (0.72–34.94)	0.1535		
							G/G	0.00	0.00	-	1.0000		
							T/G+G/G	0.40	0.12	5.00 (0.72–34.94)	0.1535		
	**rs80001089**	**T**	**0.80**	**1.00**	**-**	**0.0153**	**T/T**	**0.60**	**1.00**			**7.983**	**0.0047**
**TC**		**G**	**0.20**	**0.00**			**T/G**	**0.40**	**0.00**	**-**	**0.0120**		
							**G/G**	**0.00**	**0.00**	**-**	**1.0000**		
							**T/G+G/G**	**0.40**	**0.00**	**-**	**0.0120**		
	rs2569987	T	0.80	0.94	4.00 (0.66–24.21)	0.1792	T/T	0.60	0.88			2.904	0.0883
		C	0.20	0.06			T/C	0.40	0.12	5.00 (0.72–34.92)	0.1535		
							C/C	0.00	0.00	-	1.0000		
							T/C+C/C	0.40	0.12	5.00 (0.72–34.92)	0.1535		
***NEIL1*, chr15**													
**CE**	75,641,932	A	0.90	1.00	-	0.1328	A/A	0.80	1.00			3.672	0.0553
		G	0.10	0.00			A/G	0.20	0.00	-	0.1282		
							G/G	0.00	0.00	-	1.0000		
							A/G+G/G	0.20	0.00	-	0.1282		
**TC**	rs7182283	G	0.40	0.65	2.75 (0.88–8.58)	0.0955	G/G	0.20	0.47			1.977	0.1597
		T	0.60	0.35			G/T	0.40	0.35	2.67 (0.36–19.71)	0.6285		
							T/T	0.40	0.18	5.33 (0.62–45.99)	0.1618		
							G/T+T/T	0.80	0.53	3.56 (0.58–21.92)	0.2305		
***APOE*, chr19**													
**CE**	rs429358	T	0.65	0.82	2.51 (0.70–8.98)	0.1936	T/T	0.40	0.65			1.556	0.2122
		C	0.35	0.18			T/C	0.50	0.35	2.29 (0.44–11.92)	0.4185		
							C/C	0.10	0.00	-	0.3125		
							T/C+C/C	0.60	0.35	2.75 (0.55–13.75)	0.2566		
**TC**	rs429358	T	0.65	0.79	2.08 (0.60–7.17)	0.3368	T/T	0.40	0.59			0.894	0.3445
		C	0.35	0.21			T/C	0.50	0.41	1.79 (0.35–9.13)	0.6828		
							C/C	0.10	0.00	-	0.3333		
							T/C+C/C	0.60	0.41	2.14 (0.44–10.53)	0.4401		

UNG rs80001089 and rs2569987 variants fit well to both additive and genetic models and *POLβ* rs1012381950 fits well to a dominant model (Table 10). The epistatic relationships between *UNG* rs80001089 and *NEIL1* rs7182283 were found statistically significant (p = 0.041) in the TC of LOAD (Table 11).

**Table 10:** Analysis of gene variants in post-mortem brain tissue samples based on genetic models.

		Additive Model		Dominant Model		Recessive Model	
	Tissue type	OR (95% CI)	Fisher’s p-value	OR (95% CI)	Fisher’s p-value	OR (95% CI)	Fisher’s p-value
***UNG* rs80001089**	TC	-	0.0153	-	0.0120	-	1.0000
***POLβ* rs1012381950**	TC	-	0.0860	-	0.0198	-	1.0000
***UNG* rs2569987**	CE	-	0.0216	-	0.0351	-	1.0000

**Table 11:** Epistatic interaction between *UNG* and *NEIL1* in TC samples.

	Variations	LOAD Frequency	Control Frequency	OR (95% CI)	Fisher`s p-value
***UNG-NEIL1***	**rs80001089-rs7182283**	**0.300**	**0.000**	**-**	**0.0410**

*APOE* ε4 allele, ε3/ε4 and ε4/ε4 genotypes were not found statistically significant in CE and TC of LOAD (S8 Table). The allele frequency of ε3 was 0.65 in both LOAD-CE and -TC and 0.78 in control-CE and -TC and the genotype frequency of ε3/ε4 was 0.50 in LOAD-CE and -TC and 0.44 in control-CE and -TC. The frequency of homozygote ε4/ε4 genotype was 0.10 in LOAD-CE and -TC and not found in control and hpC-CE and -TC (S8 Table). The allele frequency of ε3 was higher in hpC samples than in LOAD (0.88 in CE and 0.81 in TC) whereas the genotype frequency of ε3/ε4 was lower (0.25 in CE and 0.38 in TC) (S8 Table). The interaction of variants with *APOE* ε4 carriers or non-carriers in LOAD-CE and LOAD-TC were also not statistically significantly associated with LOAD (S9 Table). However, *APOE* variant rs405509 was statistically significantly associated with *APOE* ε4 non-carriers in hpC samples (S9 Table).

**Fig 4:**
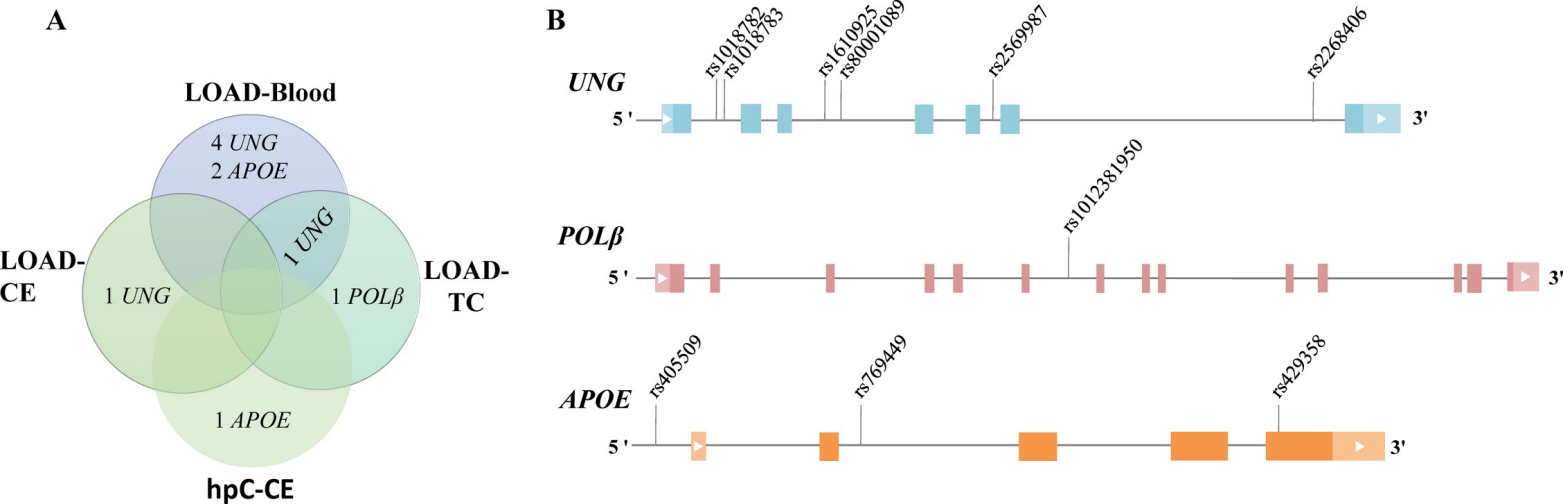
Comparison of statistically significant variants in all sample groups. (A) The venn diagram showing the common and unique variations between blood and post-mortem brain tissues of LOAD. (B) The location of statistically significant variants on their corresponding gene structure. LOAD, late-onset Alzheimer`s disease; CE, cerebellum; TC, temporal cortex; hpC, high-pathology control.

We compared the statistically significant variants between blood and post-mortem brain tissues of LOAD patients and found that *UNG* rs80001089 was present in both blood and TC of LOAD patients (p<0.05) (Table 12). The comparison of allele/genotype frequencies of statistically significant variants in all sample groups are shown in Table 12. *UNG* rs2569987 was present only in CE of LOAD patients; four *UNG* variants (rs1610925, rs2268406, rs1018782 and rs1018783) were present only in LOAD-blood; *POLβ* rs1012381950 was present only in LOAD-TC (Table 12 and Fig 4A and 4B). *APOE* rs769449 and rs429358 were statistically significantly associated with LOAD-blood (p<0.05) (Table 12 and Fig 4A and 4B). *APOE* rs405509, which is located in the promoter region, was statistically significantly associated with CE of hpC group (Table 12, Fig 4B).

**Table 12:** Comparison of statistically significant variants of LOAD, Control and hpC in blood, CE and TC tissues.

			Frequency			Fisher`s p-value			
Gene	Variant	Allele	LOAD	Control	hpC	LOAD vs Control	hpC vs Control	LOAD vs hpC + Control	Sample
*UNG*	rs1610925	TG	0.197	0.087	-	**0.0005**	-	-	Blood
			0.300	0.222	0.063	0.7190	0.3402	0.2937	TC
			0.182	0.200	0.045	1.0000	0.1745	0.5297	CE
*UNG*	rs2268406	G	0.164	0.082		**0.0052**	-	-	Blood
			0.200	0.111	0.125	0.6630	1.0000	0.4495	TC
			0.182	0.050	0.045	0.6653	1.0000	0.2204	CE
***UNG***	**rs80001089**	G	0.109	0.046	-	**0.0129**	-	-	Blood
			0.200	0.000	0.000	0.1071	NA	**0.0153**	TC
			0.091	0.000	0.000	0.3465	NA	0.0437	CE
*UNG*	rs1018782	A	0.152	0.082	-	**0.0184**	-	-	Blood
			0.200	0.167	0.187	1.0000	1.0000	1.0000	TC
			0.000	0.000	0.000	NA	NA	NA	CE
*UNG*	rs1018783	A	0.134	0.077	-	**0.0406**	-	-	Blood
			0.200	0.167	0.125	1.0000	1.0000	1.0000	TC
			0.000	0.000	0.000	NA	NA	NA	CE
***UNG***	**rs2569987**	C	0.078	0.061	-	0.5046	-	-	Blood
			0.200	0.000	0.125	0.1071	0.2139	0.1792	TC
			0.273	0.000	0.200	**0.0216**	0.4890	**0.0162**	CE
*APOE*	rs769449	A	0.134	0.026	-	**0.0001**	-	-	Blood
			0.100	0.111	0.125	1.0000	1.0000	1.0000	TC
			0.091	0.136	0.045	0.6560	0.3327	1.0000	CE
*APOE*	rs429358	C	0.189	0.061	-	**0.0001**	-	-	Blood
			0.350	0.222	0.188	0.4848	0.6041	0.3368	TC
			0.318	0.200	0.136	0.4913	0.6909	0.3522	CE
*APOE*	rs405509	G	0.472	0.444	-	0.5404	-	-	Blood
			0.450	0.500	0.250	1.0000	0.1717	0.7753	TC
			0.045	0.000	0.273	1.0000	**0.0216**	0.4062	CE
**Gene**	**Variant**	**Genotype**	**LOAD**	**Control**	**hpC**	**LOAD vs Control**	**hpC vs Control**	**LOAD vs** **hpC +Control**	**Sample**
*POLβ*	rs1012381950	T/C	0.005	0.00	-	1.0000	-	-	Blood
			0.900	0.333	0.750	**0.0198**	0.1534	0.0912	TC
			0.091	0.010	0.091	1.0000	1.0000	1.0000	CE

## Discussion

LOAD is the most common form of dementia and one of the most prevalent diseases in old age. The genetic and environmental factors that render some individuals more susceptible to LOAD are still not well understood. Effective treatments, specific risk factors and early diagnostic markers for LOAD have not been determined yet. Moreover, the molecular mechanisms underlying neuronal death in LOAD remain elusive. Several studies have demonstrated that BER defect and concomitant oxidative DNA damage accumulation may play a role in the etiology and progression of LOAD [5–17,29,34]. However, it is not known whether genetic variant(s) of specific BER genes are responsible for the reduced BER activity in LOAD patients and whether they are associated with the risk, development and/or progression of LOAD. In this study, we show that the *UNG* gene carries five statistically significant non-coding variants (rs1610925, rs2268406, rs80001089, rs1018782 and rs1018783) in blood samples from Turkish LOAD patients compared to age-matched controls and one of them (*UNG* rs80001089) is also statistically significant in postmortem TC tissue (an early affected brain region) of Brazilian LOAD patients (p<0.05). In addition, the statistically significant BER variants present only in postmortem CE (least affected brain region) and TC tissues of LOAD subjects are *UNG* rs2569987 and *POLβ* rs1012381950, respectively (p<0.05). There are no statistically significant common variants between CE and TE brain regions of the same LOAD patients. These results also reflect the difference between the germline and somatic variant distribution in BER genes in LOAD patients.

Several studies demonstrated the reduced activity and protein levels of UNG in LOAD-postmortem brain tissues [5,19,26,29]. *UNG1* (mitochondrial form) and *UNG2* (nuclear form) are generated from two different promoters, promoter B and promoter A, respectively. Rs1018782 and rs1018783 are located in the promoter B of *UNG1*. Rs1018782 is located 4bp downstream of CCAT box and rs1018783 is located within a Yi element in promoter B [49]. Kvaloy et al. screened *UNG* variants on normal and various cancer cell lines and showed that rs1018782 (position 1034) and rs1018783 (position 1082) always appear together in both normal and cancer cell lines, suggesting that they are genetically linked [50]. In the present study, *UNG* rs1018782 and rs1018783 appeared together in almost 88% of blood samples and in 33% of TC of post-mortem brain samples. None of these variants appeared in CE of postmortem brain samples. The allelic and genotypic frequencies of these two *UNG* variants are statistically significantly different (p<0.05) between blood samples from LOAD and control in Turkish population, but not between TC of LOAD and control. The epistatic relationship between these two variants (OR = 1.82) do not increase the statistically significant interaction between LOAD and control blood samples compared to each variant alone as expected, because they are genetically linked and observed together in almost all samples. Kvaloy et al. demonstrated that even though rs1018782 and rs1018783 are located in the promoter B, they do not change the transcriptional activity [50]. Rs1610925, rs2268406, rs80001089 and rs2569987 are located in a noncoding region of the *UNG* gene [49]. The effects of these variants on the expression or activity of UNG have not been identified. We performed targeted NGS analysis of postmortem brain tissues previously analyzed for UNG activity [29], and show that non-coding *UNG* variants, rs80001089 and rs2569987 are statistically significantly enriched in TC and CE from LOAD subjects, respectively. The authors demonstrated that nuclear and mitochondrial UNG activity is decreased in both CE and TC of LOAD subjects whereas mitochondrial UNG activity is decreased only in TC. However, they did not observe any change in the protein levels of UNG in all postmortem tissues, and suggested that phosphorylation of UNG protein might be responsible for the decreased activity of UNG in these samples [29]. In line with this result, we did not identify any statistically significant variant in the coding region of *UNG* gene that can affect its protein level. Our results also suggest that statistically significant *UNG* variants identified in LOAD brain tissues may not affect protein level. Although we did not identify any *UNG* gene variant that affects its function, to the best of our knowledge, this is the first study to attempt to associate *UNG* variants by deep sequencing (covering promoter, intronic and exonic regions) with UNG protein level and function in LOAD patients`postmortem brain tissues. It is noteworthy that we did not find any statistically significant *UNG* or BER gene variants in hpC individuals who do not show any decrease in UNG protein levels [29]. Very little is known about the impact of *UNG* variants on human diseases. So far, *UNG* rs246079 A/G SNP is associated with the susceptibility of rheumatoid arthritis in Taiwan’s Han Chinese population [51] and increased lung cancer risk [52]. On the other hand, rs246079 G/A is associated with decreased risk of esophageal cancer in a Chinese population [53]. In our study, rs246079 A/G was found as a common variant observed both in LOAD and control blood samples with MAF 0.36 (S10 Table)

The development of AD pathogenesis and phenotypes in NEIL1 or POLβ depleted AD mice indicate the importance of these two enzymes in AD [25,27,28,30]. Furthermore, LOAD patients have decreased NEIL1 and POLβ activities and protein levels [5,24,26,33]. However, we did not find any statistically significant *NEIL1* or *POLβ* variant that could affect their protein level and function in case-control samples, suggesting that there may be other factors such as post-transcriptional or–translational modifications responsible for the reduced activities and protein levels of NEIL1 or POLβ in LOAD pathogenesis. A recent study demonstrated that downregulation of *NEIL1* expression in the lymphocytes of LOAD patients is not due to the methylation status of *NEIL1* promoter [33]. In another study, Kwiatkowski et al. conducted SNP genotyping assay on peripheral blood samples from LOAD patients and controls, and suggested that the combination of *NEIL1* rs4462560 (p = 0.511) and 8-oxoguanine DNA glycosylase gene (*OGG1*) rs1052133 (p = 0.535) increases the risk of LOAD (OR = 2.24, 95% CI = 1.36–3.91, p = 0.041) [36]. In the present study, the NGS primers do not cover *NEIL1* rs4462560 location (Table 1, S1 Fig and S1 Table). We found statistically significant epistatic relationship (p = 0.0410) between *UNG* rs80001089 (p = 0.0153) and *NEIL1* rs7182283 (p = 0.0955) variants in postmortem TC from LOAD subjects, suggesting that the combinatory effect of *UNG* rs80001089-*NEIL1* rs7182283 variant could be associated with LOAD development. *NEIL1* rs7182283 is a common variation in blood samples from Turkish population (S10 Table, MAF.0.47) and this epistatic interaction is not statistically significant. On the other hand, *POLβ* rs1012381950 T/C genotype is statistically significantly associated with LOAD in CE samples (p = 0.0198), suggesting that the T/C genotype may be associated with LOAD development.

*APOE* ε4 is the known major risk factor for LOAD. Our study confirmed the association of *APOE* ε4 with the risk of LOAD, and *APOE* ε3 as the most frequent allele in Turkish population. The distribution of the *APOE* ε4 allele frequencies in Turkish population was reported in two studies previously [54,55]. However, to the best of our knowledge, this is the first study to sequence *APOE* gene covering promoter, intronic, and exonic regions using NGS for their association with the risk of LOAD in Turkish population. The allele frequency of *APOE* ε4 in our studied population was 18.94%, which is greater than the two previous studies from Turkey (11.4% and 17.2%) [54,55]. This heterogeneity may be due to variability in sample size, age, sex and geographical location. The *APOE* ε4 allele frequency in Turkish population (18.94%) is lower than that in Caucasian (36.7%), African-American (32.3%), Hispanic (19.2%) and Japanese (27.8%) populations [56] (www.AlzGene.org). It has been demonstrated that *APOE* ε4/ε4 increases LOAD risk 10-fold and *APOE* ε3/ε4 increases 3-fold [3]. In our study, APOE ε3/ε4-carriers were found to be higher than APOE ε4/ε4 carriers, indicating that APOE ε3/ε4 would be a main risk factor for LOAD in Turkish population. APOE ε4/ε4-LOAD association in the studied population (genotype frequency 3%) was found weaker than that in other populations. On the other hand, APOE ε3/ε4-LOAD association (OR = 4.07) was stronger compared with Caucasian (OR = 2.7), African-American (OR = 1.1) and Hispanic (OR = 2.2) cases, but weaker compared with Japanese cases (OR = 5.6) [3]. The risk of LOAD in *APOE* ε4 carriers can be increased by other genetic variants, such as *PSEN1* rs17125721 and *GAB2* rs2373115 [57,58]. The *UNG* variants do not affect LOAD risk in *APOE* ε4 carriers, but the presence of the *UNG* variants may be associated with LOAD risk in non-*APOE* ε4 carriers.

Despite the *APOE* ε3 allele being the most frequent in Brazilian population [59–62], *APOE* ε4 allele frequency was found to be higher (35%) in Brazilian LOAD patient’s hippocampus compared to age-matched control (20%), but ε4 allele was not significantly associated with LOAD risk [63,64]. Consistent with this, we show that *APOE* ε4 allele frequency (35%) is higher in all LOAD and hpC than cognitively normal controls (22%), and *APOE ε4* allele has no statistically significant association with LOAD risk in Brazilian post-mortem brain tissues. On the other hand, high ε4 allele frequency in LOAD patient’s post-mortem brain tissues were reported [65–68]. Ethnic background, sample size and post-mortem brain regions may affect the difference in *APOE* ε4 allele distribution among studies.

*APOE* rs769449 and rs429358 show statistically significant association with LOAD in Turkish population and the epistatic interaction between these two SNPs is strong with OR 6.12 (p = 0.0001). Rs769449 is in strong linkage disequilibrium with rs429358 of *APOE* ε2/ε3/ε4 polymorphism [69]. It has been suggested that the statistically significant effect of rs769449 on LOAD is probably related to that effect of *APOE* ε4 [69,70]. In the present study, *APOE* rs769449 increased the risk of LOAD in *APOE* ε4 carriers (OR = 6.09, 95% CI = 1.42–26.17, p = 0.0131), but not in non-*APOE* ε4 carriers. Rs769449 may have a regulatory effect on *APOE* by modifying the epigenetic state in the *APOE* gene region, influencing transcription levels and protein concentration without changing protein structure, and thus may contribute to LOAD [69,70]. *APOE* rs769449 and rs429358 were also found among common variants in Turkish population with MAF 0.10 and 0.15, respectively (S10 Table). Both SNPs are common in human population, except the *APOE* rs769449 that is not commonly found in the African population (S10 Table).

In conclusion, our results suggest that statistically significant BER gene variants may be associated with the risk of LOAD in non-*APOE* ε4 carriers. On the other hand, there are no statistically significant *UNG*, *NEIL1* and *POLβ* variants that could affect their protein level and function in case-control samples, suggesting that there may be other factors such as post-transcriptional or–translational modifications responsible for the reduced activities and protein levels of these genes in LOAD pathogenesis. Further studies with increased sample size are needed to confirm the relationship between BER variants and LOAD risk. This result would open a new direction for our understanding of how alterations in BER contribute to development of LOAD and also other neurodegenerative disorders.

## Supporting information

S1 Fig The uncovered primer regions in the gene structure maps. (TIFF)

S2 Fig *UNG* gene expression analysis in LOAD blood samples carrying significant *UNG* gene variants and control blood samples not carrying these *UNG* variants. (TIFF)

S1 Table The primer sequences from Ion AmpliSeq designer software. (PDF)

S2 Table Primers for Sanger sequencing. (PDF)

S3 Table Ion PGM raw data. (XLSX)

S4 Table Minor allele frequencies of the statistically significant variants. (PDF)

S5 Table Common variations in Turkish population. (PDF)

S6 Table Allele and genotype frequencies of *UNG*, *POLβ*, *NEIL1* and *APOE* in CE samples of LOAD patients, age-matched cognitively normal and hpC subjects (LOAD = 11, hpC = 11, Control = 10). (PDF)

S7 Table Allele and Genotype frequencies of *UNG*, *POLβ*, *NEIL1* and *APOE* in CE and TC of same LOAD patients, age-matched cognitively normal and hpC subjects (LOAD = 10, hpC = 8, Control = 9). (PDF)

S8 Table Allele and genotype frequencies of *APOE* ε2, ε3 and ε4 in TC and CE samples of LOAD patients age-matched cognitively normal and hpC subjects (LOAD = 10, hpC = 8, Control = 9). (PDF)

S9 Table Effect of the interaction of variants with *APOE* ε4 in CE and TC samples of LOAD patients, age-matched cognitively normal and hpC subjects (LOAD = 10, hpC = 8, Control = 9). (PDF)

S10 Table Common variations in Turkish population. (PDF)

S11 Table List of abbreviations. (PDF)

S1 Text Materials and methods for RNA isolation, cDNA synthesis and RT-PCR analysis. (PDF)
