# Supplementary figures and images for: Investigation of base excision repair gene variants in late-onset Alzheimer’s disease
Source: PLoS One. 2019 Aug 15;14(8):e0221362. doi: 10.1371/journal.pone.0221362 (PMC6695184; doi:10.1371/journal.pone.0221362)

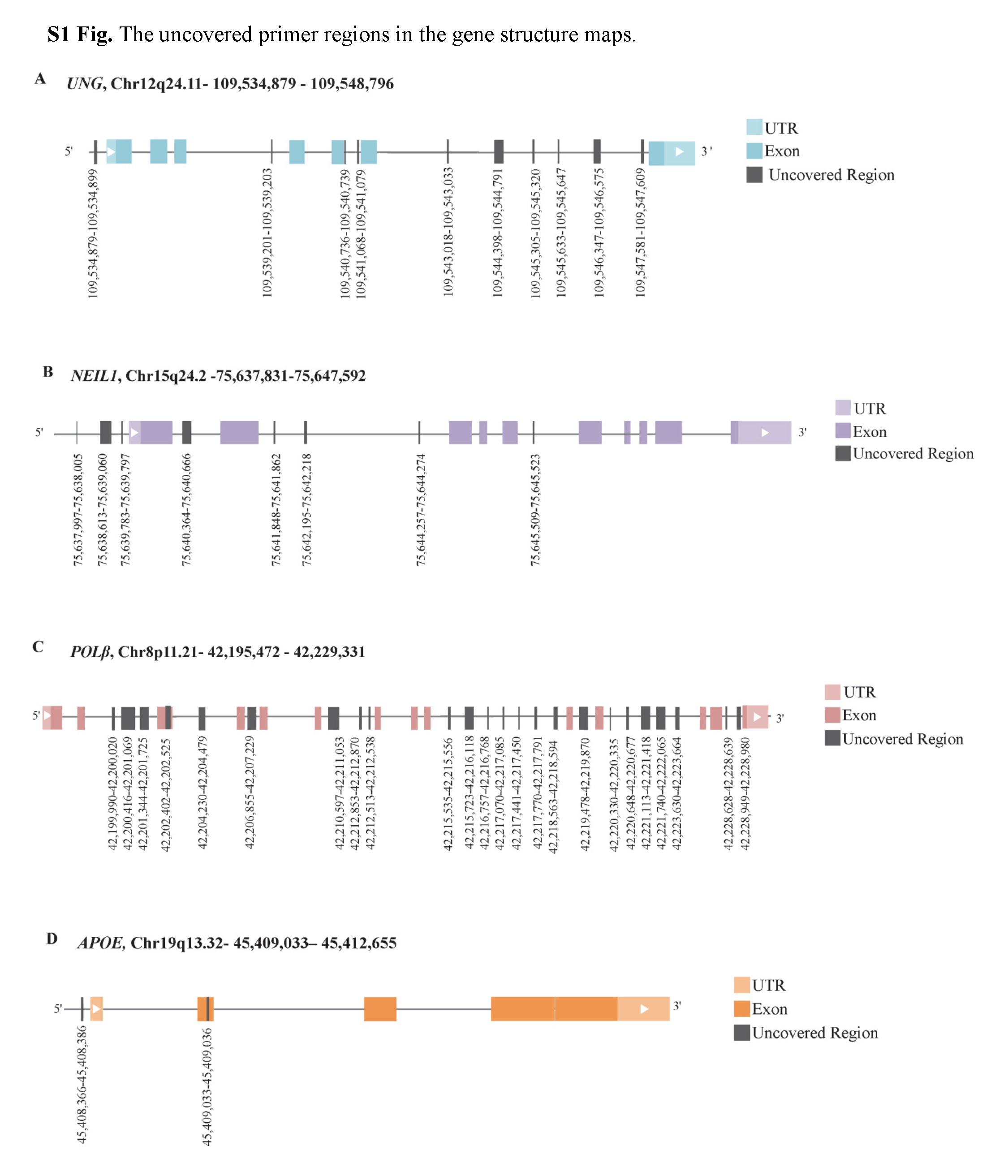

Supplement: S1 Fig — (TIFF) [file pone.0221362.s001.tiff]

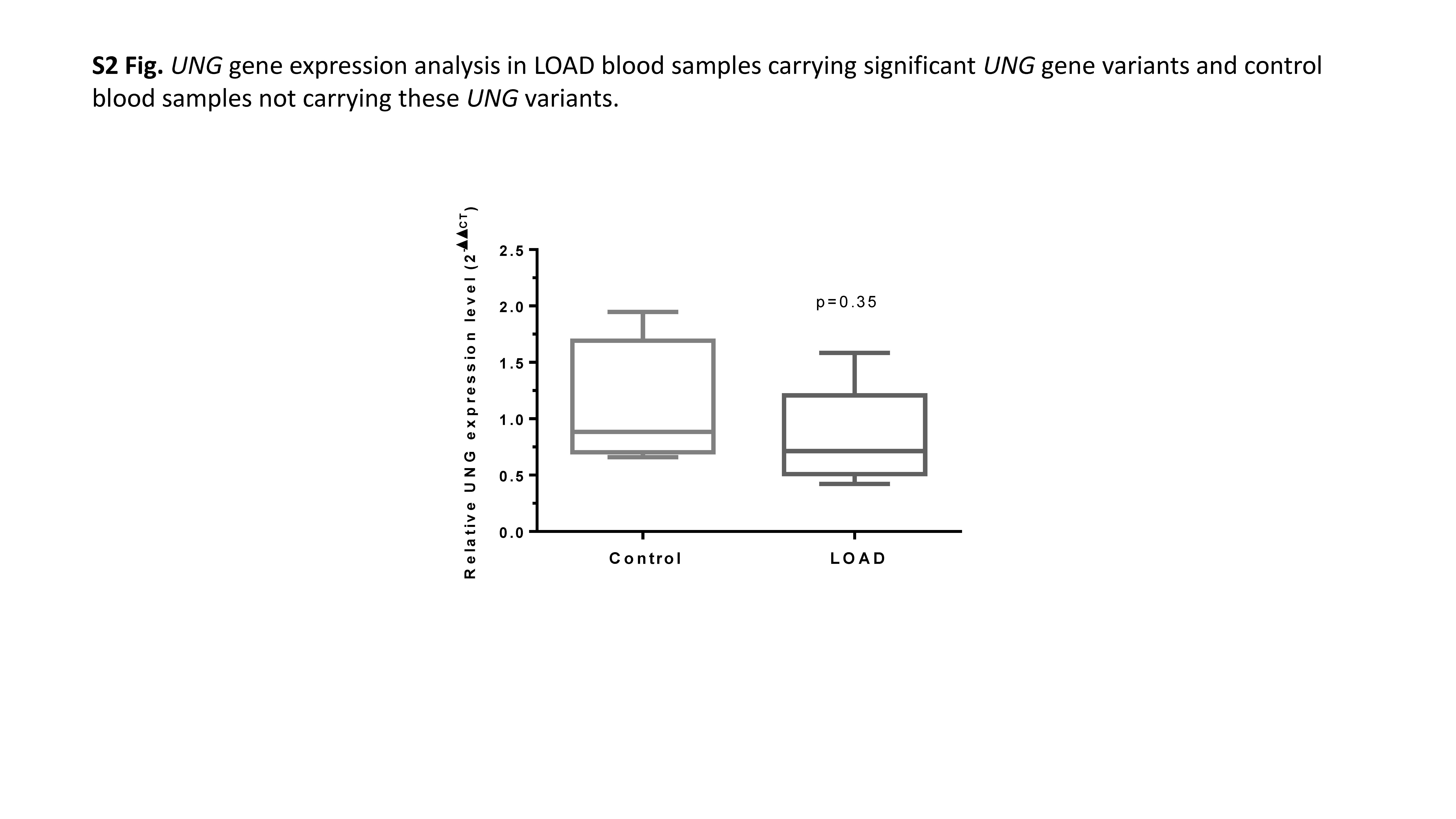

Supplement: S2 Fig — (TIFF) [file pone.0221362.s002.tiff]
