# Supplementary material for: Investigation of base excision repair gene variants in late-onset Alzheimer’s disease
Source: PLoS One. 2019 Aug 15;14(8):e0221362. doi: 10.1371/journal.pone.0221362 (PMC6695184; doi:10.1371/journal.pone.0221362)
Supplement: S1 Table — (PDF) [file pone.0221362.s003.pdf]

S1 Table. The primer sequences from Ion AmpliSeq designer software.

POLB:chr8:42,195,472:42,229,331					
Amplicon ID	Start	End	Primer Pool	Forward Primer	Reverse Primer
AMPL7160276555	42195157	42195182	Pool 1	CCAGATTTCTCTACAGCAACTACGT	GTGAACCATGCCGAGAACAGGAAT
AMPL7160090200	42195467	42195489	Pool 2	GGTTGGCAGGTGTACTAACACG	GAAGACCTGGAGTTTTGACCTAAGATATTA
AMPL7158705911	42195748	42195774	Pool 1	GGGAAAGGATTCCAGATAAACACTGA	AGTGTTCAGAACCAGGGACTAGA
AMPL7155757227	42196060	42196083	Pool 2	CAAGTCCTGGTACCTCCTTCAAG	CTACCCTACCCACTGGACTGT
AMPL7157138981	42196230	42196255	Pool 1	CTTTCTTCTTTCCTTCCAGCCTCTT	GCTCGGGTGAACAAGAACCA
AMPL7157138982	42196459	42196482	Pool 2	CCCTTCCAGAAAACAGTTCTCGT	CAGCCTCGATTCTTGCTTTTTCC
AMPL7157138983	42196664	42196686	Pool 1	GCCCAGTGGATATTTGGTCCAT	CCTGGTGAGATGAACAAGGACA
AMPL7157138984	42196968	42196992	Pool 2	CTGATCCTGATCCTTGTTCCTTGT	TGTGGACCATTTGAGTTTAGGAGTTG
AMPL7157138985	42197112	42197134	Pool 1	GGTCTCGCTGTGGTATGATCAT	TTTGCTTTTAAGAGCTGAGGGACTT
AMPL7157138986	42197378	42197400	Pool 2	GGTGTCAGCTCTCCTTAGCCTA	GGACTGGGACTTCTTGAGTAAATGAT
AMPL7157138987	42197508	42197533	Pool 1	AAATCTTCATTCCTTGCCTGGACTT	GCATGAAACAAAGTTTTGACCATTTTGAC
AMPL7157138988	42197811	42197838	Pool 2	GGAAAATTCCACATCCGATCTCATGTA	AGTCAAGAATGGCAAGATTGCCA
AMPL7157138989	42198108	42198133	Pool 1	GCCCTTAGCCCTCTTTTCTTACTTC	CAATCAGCTCTGAACTGTGTGGA
AMPL7157138990	42198247	42198269	Pool 2	TATCCCAAGTCAGCTCCCTTCT	GTAGGGAATCACCTGCTTTTCCA
AMPL7157138991	42198563	42198588	Pool 1	TTGCTTAACGGGTATGGTATGAGTG	CACATGACTGATGATGAATCTGAAAAGAC
AMPL7157138992	42198872	42198894	Pool 2	ACTGTGTTTGCCTGCTCCTTTA	TTCCCACTGCAGTTCAGCATAA
AMPL7157138993	42199162	42199184	Pool 1	TGTGGAGGTGGGCAAGAATTAC	TGGAAGATAAAGATGCTTACAAGAAACAGT
AMPL7157138994	42199458	42199488	Pool 2	CCTTCATCATGTCTGAAGTTATGCTATTCT	CGATAATACAATGTAGAAAGCATTGATGGT
AMPL7157138995	42199658	42199680	Pool 1	CCTCCATCCAGGTCACCAAAAA	ACACACCCAAATAGAGGATGATAGTTCATA
AMPL7157138997	42199989	42200021	Pool 2	TATATGAACTATCATCCTCTATTTGGGTGTGT	GGACTTTGTTAAAGTTGTTAAGAATAAGGCCTA
AMPL7157139302	42200075	42200107	Pool 1	TACCATCTTGTCATCTTTAAGTTTCATTTGGT	CACACGCAGCCCTTGGATA
AMPL7157139303	42201039	42201070	Pool 2	AAAAAGTAACATAGGTATCCATAAGCAGAT	CTGGCCTACGTCTAACTTTTAAAGTATCT

				C	
AMPL7157139304	42201702	42201726	Pool 1	CTGTAATCCTAGCTGGTTCACACC	CTGGCCTAATTTTCTACCTCCATATGG
AMPL7157139003	42202018	42202039	Pool 2	GGTGGTCTGTCAGCTCATCTG	CAAATATCTATGCTTATGCTTTCACCAAG A
AMPL7157135855	42202169	42202199	Pool 1	GGTGAAACATCATCTCCACTAAAATACAAG	CATCAAGGCCTTAATTTTTCTTAAACTGG A
AMPL7157139005	42202500	42202526	Pool 2	CACACAAAATAAAGAGTGGAGCTGAA	GTGCCACTTGGTCACTCTTCTTAA
AMPL7157139006	42202795	42202823	Pool 1	ACATGTACTCACGTTGAATTTCAACTTG	CAAAATAATCTGTTCCCAAGCATTTCAGA T
AMPL7157139007	42203048	42203076	Pool 2	GGGTCACATGCCTCGTTAATTTGTAATA	TGGTCCTTAAATTTAAGGAACTTTGAATC A
AMPL7157139008	42203321	42203354	Pool 1	CTATCTATTGACAATCAGATTGTTGTCTCATC T	GGCTCAAGGCAGTGGATCAC
AMPL7157139009	42203411	42203442	Pool 2	TTTGTGAAAGATGTCAAATTATCATGGCTTT	AGGAGGTGATAACTATCAGGACCTG
AMPL7157139010	42203735	42203756	Pool 1	CAGCCTGAGATGCCTTTTGAG	CTGCAGCAGTAAGCTGTGATTATG
AMPL7157139011	42203855	42203879	Pool 2	TAACCTTCTCAGGAGATGAGCCAT	GCAGTTATGGTCCAGTCATGGT
AMPL7157135699	42204046	42204073	Pool 1	GCATGCTCCATCACACTCAGATATTTT	GGTAATGTTATGACTCTGAAGGGCAA
AMPL7157139013	42204451	42204480	Pool 2	ACTTTAATTCTGAGTTTCTGTCTTGGTGA	AATTACTGAGGAGTAATTCCAGAAATGG
AMPL7157139014	42204716	42204748	Pool 1	TCCTTTTCACAATTATTGAAGAATCACAGAG T	CTGGAAATTTCAGATTTCTAGAATTACTG AGAA
AMPL7157139015	42204987	42205020	Pool 2	TCCTTCTTAGTAACGGAAGTAATAGATTCTT CT	AGATAGAAATGAACAAAAGATTTAGGAA GCAGA
AMPL7157139016	42205228	42205250	Pool 1	ATGAGCCTGAGCAGTTTCCTTT	TCTTTCTCCACACTGCATGTATTTGAT
AMPL7157139017	42205504	42205532	Pool 2	ACCAAAGTTGCAAATTAAACAAGACACT	AGAGCAGAGATCCTGTCTTTTCATTTT
AMPL7157139018	42205743	42205768	Pool 1	TTTTTGATCACCTGTATGCCAGTCA	CCCTAGAGTTTCTCATTCACCATGT
AMPL7157139019	42206030	42206056	Pool 2	CCCTGAGAACTTAGGCATATTCTTGG	CCAGTGAAAATAAATTCTCCCGAAGACA
AMPL7157139020	42206326	42206353	Pool 1	TTCTCAGAGTTTCCCTTTTGTCATTCA	AGTTGCTAAAAACTCATCAATCTTTTCAG C
AMPL7157139021	42206514	42206544	Pool 2	ATTTCTAATTTTCCATGTAGCCTGGAGTAG	AACGATCTTTCAGTCAAAGTTTATAATCT CT
AMPL7157135716	42207201	42207230	Pool 1	CGGCCATCTTTTAGAATTTCTATGTTTAC	CCTGGGTGACCCACACAAAATT
AMPL7157139022	42207351	42207381	Pool 2	ACTCTGTTATTCACCTATTACTCCTTAGGT	AACTAACTGACGATTGTGTCTGCTTAA
AMPL7157139023	42207565	42207587	Pool 1	CTCGAGTTAGTGGCATTGGGTA	ATGCTCAGAGAAACAAAAGAGGCCAA
AMPL7157139024	42207855	42207880	Pool 2	CCATTTCCTCCGTGTGCATCTATTT	CCATAAAGATGAGAGCACTAAAGCCTT
AMPL7157139025	42208156	42208186	Pool 1	TTTTTGACACTGGCTGAAGTAAAGATTAAC	GTACTAAGGCAGGAGAGTCACTTTAC

AMPL7157139026	42208413	42208442	Pool 2	GTGTCTTATTCTGCTATCTAGGCTAGAGT	TCAACTTTCAGTTCTCTTGCCTAGAAAA
AMPL7157134131	42208677	42208702	Pool 1	ACCACAAGGTGTTTTAGAGAGCATC	CAGCATAGAAGAAACAGAACTGAACTC
AMPL7157139027	42208763	42208787	Pool 2	GCAGAGCCAGATTTGAACCCATAT	GAAGGACACTGAAACCTAGAAAGATGAA
AMPL7157139028	42209064	42209086	Pool 1	GCACCAGCTAGATTTGTGACCT	TTCAAGAGAAGAGAAAAACTAGGATGCA T
AMPL7157139029	42209377	42209399	Pool 2	CATAGCACCTCATACCGTCACT	GGCCATTATGTGTCAGATACTTTCCTG
AMPL7157139030	42209679	42209706	Pool 1	CCCACTTACATTCTTATTCCTGCTACA	TCAACATTGGGTGAAACTTGGGA
AMPL7157139031	42209953	42209981	Pool 2	GCAGCTCTTCTTACTATTAAGACATGGT	GCCCTGGTTCTTGGTCTTCTTA
AMPL7157139032	42210255	42210288	Pool 1	CCATCATAAACATTTTTAGGTTTGTAACTAA CT	AGGTTGGTTCTTGGTCTTCTTCTTTTT
AMPL7157139305	42211021	42211054	Pool 2	GGTTATTAAAGGAATTTTTGCTTTCAAAGAA AA	TAAGGTCATCCCACTCTAATAAAGTAGTG G
AMPL7157139038	42211244	42211266	Pool 1	TTTTGGAGACTGACCTGGCTTT	AATACCTCCAAGAAACCCACAGATTC
AMPL7157139039	42211547	42211576	Pool 2	GACAGCTTATATGAGAGAATTGATTGGCT	GCATGCTTCAAAAGCAATTCCTACAAG
AMPL7157139040	42211846	42211873	Pool 1	CTCCAGAGGGAATGTGTATATTTGTGG	TTCTGGGCAGACAGAGCAAGAC
AMPL7157139041	42212037	42212070	Pool 2	CAGGTACAGTAATAATTTTCCTTTCAATGGA AT	CATTAGCACTGAGTAGTAGATTTGTGCA
AMPL7157135868	42212354	42212374	Pool 1	CCGGCTCAACAGGATTTTGA	AGAATCACACCAAATTTTACTGACTATAG TGG
AMPL7157139043	42212506	42212539	Pool 2	AAATTAAACCACTATAGTCAGTAAAATTTGG TG	GAGCTAGATAAAAGTGACTCTTCAGGG
AMPL7157135869	42212850	42212871	Pool 1	CTGGCCCTGAAGAGTCACTTT	CAAGTGTCAAAAGAAAATCTGCCATCTTA
AMPL7157139046	42213047	42213076	Pool 2	TGAAGATAAATTGAACCATCATCAGCGAA	CCCTGCTAAAGTCAAAAGAATAAGGGA
AMPL7157139047	42213257	42213287	Pool 1	TGCTTTCACTTTTGCTTTCTAGTTTACTTG	GTTCCCAGACTATATCTGGCACA
AMPL7157139048	42213552	42213582	Pool 2	CTACTAGGCATGTTTCTATATCAGCATCAA	CACCTCCATTTACTACAGAAATGCCA
AMPL7157139049	42213782	42213804	Pool 1	TGAGAGACCTGGATGGTATGGT	GGGTTCTTTCTGAGATAACAAAAATGTCC T
AMPL7157139050	42214025	42214048	Pool 2	GCACAGTGTAGGAACCATCACTA	AGTTTACCGCTCTTATATCCTGCAAAATT A
AMPL7157139051	42214260	42214288	Pool 1	GTTGTCTGAGTTTATGTTGCAGGATAGT	CAGATCATTCTCACAAATCAAGGCTAG
AMPL7157135758	42214528	42214552	Pool 2	CCCAATTTTGCTGTTGTCATCTCA	ACCAAGTGACACTCTCAATTCTAATCAAA G
AMPL7157139052	42214724	42214753	Pool 1	GAGATGTTACAAATGCAAGTAAGATGTGT	GCAAAAATAACCCAAGATTAGGAAGTAT GT
AMPL7157139053	42214881	42214903	Pool 2	TTGCTACAGTCTGTGGCAGTTT	GGAAAAAGAAAGGTAAATTAACAAAACT ACCCA

AMPL7157139054	42215187	42215211	Pool 1	CCTTTTGGCTGGATGATAGTGAAG	TCTTATAGAAAACTTGGGCAGTTGGG
AMPL7157135871	42215535	42215557	Pool 2	GCCCAACTGCCCAAGTTTTCTA	GCGCCTGGCCAAAAGTTAATATTTT
AMPL7157136570	42216086	42216119	Pool 1	TGTTAGAGTAGATAGAGTAGATACCTGAACTGA	CTCCAACTCTAGATTCTTACAGCAGC
AMPL7157139307	42216151	42216174	Pool 2	AGTAGGTGATGGCCTATCAGGAA	GCTGTGCTTCCAAATCTACATTTTGAAAA
AMPL7157139309	42216430	42216458	Pool 1	CAAATAAGAATCTCCTTAGGTGGGAACT	ATGTAACTTTATGAAAGAAAGGGAGAGAAG
AMPL7157139310	42216736	42216769	Pool 2	AGTATACCATCTTTATTTCTTTCTTCTCTCCCT	CCCAGGCAACATAGTAAAACCATG
AMPL7157139311	42217058	42217086	Pool 1	TTTTTGGTAGGGACATGGTTTTACTATG	GCGGACTGGATAAACAAATGAAGTATG
AMPL7157139313	42217191	42217218	Pool 2	TTTTTGTAGCTGTGTAACATTCAGTTG	AAAAAGAAGACTAGACTGAATGTGTACTG
AMPL7157139064	42217427	42217451	Pool 1	GTTTCCATGGATGTCCAGTACACA	CCAAATATTTAAATATAAAGAAGGCCAGGCC
AMPL7157139314	42217770	42217792	Pool 2	CGGCCTGGCCTTCTTTATATTT	CATATGTAAAAGCATCCACTATAGTGCCT
AMPL7157139067	42217928	42217957	Pool 1	GAAAGGGAGGAGATAGATTGGATTCTTTT	GAGAGATCCAGGCACTCTCTTTTT
AMPL7157139068	42218229	42218259	Pool 2	TTGAGTTCAACCATAAGAATTGAAATGAGT	GGCATTAGATATACATTGCATACAACGAGT
AMPL7157139070	42218566	42218595	Pool 1	TCGTTGTATGCAATGTATATCTAATGCCA	GACAGAAGACACATATGAGATTGTCCAT
AMPL7157139071	42218745	42218767	Pool 2	GCTTGGTTCCATTTGCCCAATT	CAGCAACTCATGGAAGAATAATAGGTATCC
AMPL7157139072	42218997	42219026	Pool 1	TCTGAAGAGCTTTGTACTGATTGAATTCT	TGTTTCACCTTCCAAAGGCATATATTCTAA
AMPL7157139073	42219219	42219248	Pool 2	CTTCTATTAGAAAACCTGTGAAGGCAAAA	CGGCCATCTCTATGTTTTCTAATGT
AMPL7157139315	42219840	42219871	Pool 1	GAAAACTCATGTTAAAAATGTTTTCTTCCCG	TTAGAGACACAGTCTTGCTATATTGCAC
AMPL7157139316	42219991	42220017	Pool 2	CCATGATTGTACCTGTGAATAGCACA	CCCTGCTATTATTTTTCTTTAAAGTCTCATG
AMPL7157139317	42220305	42220336	Pool 1	GAATTACAGTCACCAAATAGAGTATCCATGA	GGCATCCTGCTGATTATTTTTCTTTCTTTT
AMPL7157136859	42220648	42220678	Pool 2	AAAAAGAAAGAAAAATAATCAGCAGGATGC	CCATGCCCAGGCCTAATTAACT
AMPL7157139077	42220729	42220757	Pool 1	TTCTCCACTTGTAAATAACACGTGTCAT	AGCAGATTATGCCTAATTAACCCATTCTTT
AMPL7157139322	42220818	42220839	Pool 2	CAGCGGATTACAAGGTCAGGT	AAAAACTTACTGTGTGTCAAAGTCATTACTG
AMPL7157139326	42221391	42221419	Pool 1	TGATTCTGTATAAACACCAACAGGAAGG	ATCTTAATTCTAGGCTGGGCACG
AMPL7157139327	42221397	42221421	Pool 2	TGTATAAACACCAACAGGAAGGCA	AAAAAGGAACTAAGAATCTTAATTCTAGGCT
AMPL7157139084	42222043	42222066	Pool 1	GGGCCTCTTCTTTCTTAAGGTGA	ACTAATTTTAGCACATCCACTCTCCAAA

AMPL7157139085	42222128	42222152	Pool 2	CCGGGTGATATGACTGAAAGGAAA	CCACTGTGCCTACTATTTTAACCATTTTT
AMPL7157139086	42222314	42222341	Pool 1	GGGTAATAGGGAGTTCCTGTTTAAATG	TTGTATGTGTGTGGGTTAAATATACACA
AMPL7157139087	42222617	42222648	Pool 2	AAAAATAGATGGCCAATAGATGGTAAATTGT	AAAGTTCGTTTGGAAGCATTGTTAAAGT
AMPL7157139088	42222712	42222739	Pool 1	TTAGTGGGCTGTGAAAGTGTATTTTCT	CCAGACGTAGCAAGATACTTTTCTACTG
AMPL7157139089	42223018	42223048	Pool 2	TCCTAAAAACAGTATCTGCTAGACAAGTTG	GCCTGGCCCTTTTACCTTGTATT
AMPL7157139090	42223286	42223313	Pool 1	AAACCAACAGAAGCTATAGGAATCCAG	CCAGCCCACTAGATTATACTTTTTCCTTTT
AMPL7157139092	42223636	42223665	Pool 2	GAAAAAGTATAATCTAGTGGGCTGGATGT	CTGAGATGGATTCCTATGTCGCAA
AMPL7157139093	42223939	42223969	Pool 1	AGATTCAGGTGACAATAAGAACAAGAAACT	ACTCAGGCCTTTTAGAAGTAAATCCAC
AMPL7157139094	42224232	42224259	Pool 2	GCTCCATTACTAATACTCTGTATGGCA	TGCTCACTCTCCTCATTCTGTTCT
AMPL7157139095	42224544	42224570	Pool 1	GAAGGGATAGAAAGAGAGAGGATGGT	CCCTAAGTCTCATGTTCTCAATGAGG
AMPL7157139096	42224846	42224872	Pool 2	TGCAGGAGGCAGTATTAAATAGTGTG	ATGGAATGAGAATGGTTTATTTCACTACC T
AMPL7157139097	42225029	42225057	Pool 1	GCTTCTAAAAGGAAATTGGCTGCAAAAT	CAAAATACTGAAATCTGAATGGCACTCA
AMPL7157139098	42225327	42225353	Pool 2	AGTTTCCTCTGCATTCAACTATTGGT	TCATGGCTTTGTTCCTTTCCTCAA
AMPL7157139099	42225600	42225624	Pool 1	GCACTTCAGAAAAAGTGCAGGAAA	CCTCAAGAGTGGTCAAGTTAACACA
AMPL7157139100	42225904	42225934	Pool 2	TTTCTATTTTGCTGAGTGAAAAAGTATGGC	CTAGAAGGGAGAAAGACACGGAAAATAA GA
AMPL7157139101	42226171	42226199	Pool 1	AATTACCTGTGAAGGAAGAAAGTGATCC	ACAGACATCAAAATAAACAGAAGAGAGC AA
AMPL7157139102	42226431	42226460	Pool 2	TTTGTTTTCTTCTTCCTGCATAATCATCG	AGCTCTAGAATTCTGAGCACTTTTAACTT
AMPL7157139103	42226621	42226650	Pool 1	ATAGAAAGGTAGGGATAGTGTATTGCTCA	AAGAACATATGGCTCTTGGGAGTAAAAA
AMPL7157139104	42226916	42226946	Pool 2	CCTGTTTGTATCTTGGAGTTCACATTCATA	CAAAATACCAAAGATAAGCCTAAGTGGT CT
AMPL7157139105	42227217	42227243	Pool 1	GCAGTCTACCTCATGATAGTCTTCTC	GAAGGTTTGCTAAACCAAAGTATCTTTGA A
AMPL7157139106	42227519	42227549	Pool 2	GTGTGTATTAGAGATCATCTCTCATCTGGA	GTGGCACTGAAAACAATGATACTTGAG
AMPL7157139107	42227799	42227828	Pool 1	TTTTGCTGTTGTTTAATGTACCTCTAGGA	GCAAGCCAAAGGAATTAGAATCTGAATT TA
AMPL7157139108	42228103	42228125	Pool 2	TCAGTGTGCATACCAGCAACAT	CAACTCGGTTCTTGGACTAGCA
AMPL7157135895	42228414	42228441	Pool 1	CCATGGTTGGATAAGCTTGTTCTAGAT	AAAAAGACACTGACTACCTCTATCCT
AMPL7157139333	42228619	42228640	Pool 2	CTCCTGGGAGAGGATAGAGGT	AAAAAGAAAATGGTAACCAAGTCACTAA AAA
AMPL7157139335	42228950	42228981	Pool 1	TTTTTAGTGACTTGGTTACCATTTTCTTTTT	GGATACAGGCCTCATTCGCTC
AMPL7157139113	42229133	42229155	Pool 2	CATCCAGTGGAAATACCGGGAA	GGTAATGTGGTTTATAGCATAGAGCAGA AT

UNG:chr12:109,534,879:109,548,798					
Amplicon ID	Start	End	Primer Pool	Forward Primer	Reverse Primer
AMPL7160090178	109534875	109535223	Pool 1	GTTTGTTTTTGAGATGCCTCGGATT	CTGCTCTTTGGACAGGCTCTTA
AMPL7155382774	109535168	109535520	Pool 2	GCCTGACCAGTCTTCTCTTCTTG	GGAGAAAAAGGAGTAGAGCGTCTT
AMPL7157137438	109535469	109535751	Pool 1	CCTCCTCAGCTCCAGGATGA	ACACGTGGAGGCTATTTGGAAA
AMPL7157137439	109535699	109536073	Pool 2	GCGCCTCTGACTCGGTAAAC	GGAATTGGGAATTAGGTTCTAAACTGGAG
AMPL7157119888	109535786	109536146	Pool 1	ATGCTAAAGGGCCAGCCAAT	GCAGAAGACGCCCATTTGTG
AMPL7155381294	109536094	109536444	Pool 2	GCTCTTACTGTCCGCTTTTGCT	CCTTGATAAAATACGGTTTCCCGAACT
AMPL7157137440	109536382	109536744	Pool 1	GCTTTGGAGAGAGCTGGAAGAAG	CTCAAGCCAGGTTTCATTCAGGA
AMPL7157137441	109536689	109537044	Pool 2	CAGGAAAGGCTGGCGTAGTAAA	GGGTGGATAAACAGTGTAATGCTTTC
AMPL7157135503	109536981	109537218	Pool 1	ATCTTTAAATCAGCTAATGGGATTTGTTGC	CAAACCTCGGGATTTCAGTGTTTTATTT
AMPL7157137442	109537158	109537414	Pool 2	CTGGAGTTAAGCCACAGTCCAT	AAAAGATTTAACTGCTTTGTGTGTCAGTT
AMPL7157137443	109537353	109537698	Pool 1	CGGTGTCAGATGTTATGGTGGT	ACCAGGTTTCATTGGTCTTGAACTC
AMPL7157137444	109537566	109537940	Pool 2	GGCTCTCATCCATCTTGTGTTCA	CGTGGCCCTATTTAAAGAGGGTT
AMPL7157137445	109537883	109538162	Pool 1	CGTGGCTTCTCACTGTTGAACTAT	GGGTAAGAAACCTAAAAGTCCAGTGT
AMPL7157137446	109538104	109538471	Pool 2	TCTGAGCTGCTACGATGCATTT	GTTTTTGAGCATTCCCTCAAAATATTCCTT
AMPL7157137491	109538396	109538655	Pool 1	TGCCCTATTCTGAATACCAGTAATCAGTA	TGACATACTCTATCTTTCAGAATCCAGCT
AMPL7157137492	109538596	109538916	Pool 2	CCCAAAAGCCTCCTGGTCAT	AAAAAGCAATTCAGATTTCCCACCA
AMPL7157137493	109538867	109539228	Pool 1	GGGAGCAGACACCCTCATA	CCCAGTTACTTTTCGATGAATTAAAGC
AMPL7157137451	109539184	109539554	Pool 2	CATGCCAGGCCCTAAATTGC	ACTTGTAAAGATATGTACCTAGCTGCTTTT T
AMPL7157137452	109539328	109539649	Pool 1	TAAGGGAGGCCGTGTGTCTAC	CTGCCTCAAACAAACATAAACAAAACAA AT
AMPL7157137453	109539581	109539786	Pool 2	CAACTCTGCTATTAGCAATCTATGCCAT	GGCCTTTGAACACTAAAGCAGAG
AMPL7157137454	109539726	109540084	Pool 1	GACAGGATCCATATCATGGACCTAATC	GGCAATGTCTGCACATTTTTCATTATCA
AMPL7157137455	109540023	109540357	Pool 2	CTACCACTGGAAACCAGGAGCAA	GGACAGTTTTCTGTCCTTAGCATATTTATG
AMPL7157137494	109540295	109540460	Pool 1	CCACTGCTTTTGGAGGATTTGG	GCCAAAGACCTTTACTTTAGTCTGTACT
AMPL7156710119	109540394	109540757	Pool 2	GTTGATTTACTTAGGTTTTCCAGAGTGC	AAAAATCTAGCAGTCGCTGGC
AMPL7157137495	109540719	109541090	Pool 1	GTGGGCCAAGCAAGGTAAGCC	AGCAGCCACTACCATCAACAAT
AMPL7157137459	109541060	109541250	Pool 2	CCCGACTCCATTGTTGATGG	AGAAGGAGAACACCTATACACATGAGAG
AMPL7157137460	109541190	109541562	Pool 1	GCTGATTTGCCTGAGCCTACAT	CCCTTATAACAGGCCTAAGGGAAAATAAA A

AMPL7157137461	109541498	109541807	Pool 2	TGTTTGGTCCTGGAAAATCCATGT	TTTGTGATTGAGTGGAATTCTTTGTGTTTT
AMPL7157137462	109541742	109542116	Pool 1	ATGTTTCTGGCTTCCAAGAAATTTG	CTTTGGAGGTTGAGGAGGACAG
AMPL7157137463	109541833	109542205	Pool 2	CAGTGATCATTATTGTTTTGTTTCTGGGT	GGTCGTGTTGGGTATCTGCAAT
AMPL7157137464	109542148	109542469	Pool 1	CGGCCTATCGGTGATCATTATTAACC	AGCCTCCATCTCTAGTCCATCAC
AMPL7157137465	109542412	109542728	Pool 2	GGCATGAGTCACCATCATCTTTCT	AGTGCTCAACCTAGTACCCACA
AMPL7157137466	109542667	109543041	Pool 1	GGGTATCAGAAATGTGCACGTATATTTAAC	TCTGCATGCAAAGTACTCAACCT
AMPL7157137468	109543012	109543381	Pool 2	CCCGACCAGGTTGAGTACTTTG	GGAAAATGTTGAATTTGGCATGTTCATG
AMPL7157137470	109543316	109543658	Pool 1	TCTCTTCTGATCTATAGAGTGCCTTGT	ATCAGAACCCTCCAGGCAAGAT
AMPL7157137472	109543604	109543879	Pool 2	GACCACCTTTGCCTTCATTCAG	GAATCCCAGAAGAAAATGACCTCTACA
AMPL7157137496	109543819	109544110	Pool 1	CTGCCTTTGCATATCCTGCTTTC	CTACCTCTACCCTCAGTAGCTGTAC
AMPL7157137497	109544055	109544426	Pool 2	AGCAGCTTAGAGTTGCAGCA	CGGCCAGAATATTATAATCTTATGGGAC
AMPL7157137498	109544759	109544904	Pool 1	TCTTTTCTATGTTTAGATACACAAATGCCT ACC	TGGGATAACCTACCACTACACACC
AMPL7157137499	109544845	109545139	Pool 2	GTAGACCAGGAGCAATAAGCTACAC	CTGCTACTCAGGTGACTAAGGTG
AMPL7157137500	109544978	109545328	Pool 1	GCATCCCTGTCATTAGGTGATGT	AAAAAGAGGAATAGTCAGTGGCC
AMPL7157137501	109545300	109545658	Pool 2	ACACCTGGCCACTGACTATTC	AAAAATCTCGCCACAAAAATGGAAT
AMPL7157137502	109545625	109545750	Pool 1	CGGCCAACTATTCCATTTTTGTG	GCCATTTACTTTCCATTTTGTGCCTTT
AMPL7157137503	109545683	109546057	Pool 2	TCTTCCTTCTTAGGAGCTGTAAGACTATTC	TCATAAGCCAGGGACCAGTTGTA
AMPL7157137504	109545997	109546370	Pool 1	AGTGTGTCAGTCCCTTTATATGTACCT	CCACCTCCTAGGCTCAAGTTACT
AMPL7157135550	109546551	109546823	Pool 2	TCAGTCTTCTGTGTAGCTAGGACTG	GGGCAAAACAAAGCTTCTTTTACAATGA
AMPL7157135551	109546757	109547023	Pool 1	AGTACAGTGTTCAATGAGTTACATGAGG	AGGACACCATGATGCATACACAC
AMPL7157137505	109546963	109547283	Pool 2	CCCATCCATTGTGTTAATAAGTTGAGG	AAAAGGAAGGACTGTGGCTGTT
AMPL7157137485	109547236	109547610	Pool 1	GGGAATCGCACAGGGTCTTA	GTGGCAGATTTGGGACTTTTTAAATTTTT
AMPL7157137486	109547582	109547815	Pool 2	AAAAATTTAAAAAGTCCCAAATCTGCCA	CGTTAACAGCAGCTTCTCAAAGG
AMPL7157119889	109547759	109548078	Pool 1	CTGGAAGGAGCTGTGATCATCAG	CCCTGTTTCACCATAAAGGCAAG
AMPL7157119890	109548024	109548389	Pool 2	GGTCAAATACTCAGTTGGCTCTCT	TCTCGGTCTCTTATCTGATAAAGCTCA
AMPL7157119865	109548325	109548515	Pool 1	GCTCAATTTCCTGATTGTAGTAGAGGT	GGACATAACCCTAGGAGAAAATCACC
AMPL7160276503	109548452	109548821	Pool 2	GGAAGAGGAGAAAAGGGAATTTTGTCT	CACAAAGGACACTTGCACAAAAC

***NEIL1*:chr15:75,637,831:75,647,592**					
Amplicon ID	Start	End	Primer Pool	Forward Primer	Reverse Primer
AMPL7160266878	75637650	75638024	Pool 1	GATGCAATTCAAAGTACAGAGCAGTG	AATATTCAGGGATACTTCCTAGGTGCT
AMPL7160266879	75637985	75638290	Pool 2	CGAAGTACAGAGCAGCACCTA	GCGCCTGGCTGGATTATATCTTTTTAATA A
AMPL7160266880	75638228	75638521	Pool 1	GGAGAGGGAAGGTGGTTTCATC	CAGGCTACAATCATGGGTCATTG
AMPL7160088421	75638380	75638637	Pool 2	TAGTGAGACCACATCCTTACAAAACATG	GCCCAGCCAACAGTAACTTCTTAA
AMPL7158763775	75639030	75639293	Pool 1	AGACAAATCATGACATTTCATACCCAATACT	CTGGAGTATAGCTGCGGGTTAG
AMPL7157141564	75639238	75639567	Pool 2	CCTGGGTTTGCTTCTCTGTCTTC	TTAAGCACCAACGAACAGTCAGA
AMPL7157141565	75639513	75639803	Pool 1	CAGGCCGTGATGATGTTTGTT	GTGGCGGAAGAACCTGACTT
AMPL7157141567	75639776	75640069	Pool 2	GCAGTGGGAAGTCAGGTTCTTC	GCTCATCTTCTCCTTAGCCCTACA
AMPL7155948367	75640017	75640384	Pool 1	CGTGCAGGATCGGGTTGT	GGGTATCGACTCCTCACCTC
AMPL7157141594	75640649	75640928	Pool 2	GGCCGAGCTTCGCTCTTT	CGGCTGCAGCCTTACTTCTT
AMPL7157141595	75640876	75641194	Pool 1	CGGAGATGAGATCCGTGAGTGA	CGGTGTGGACTTTGTGAGGTAG
AMPL7157141569	75641137	75641346	Pool 2	CTCCGAGTTCTCCTCTAAAAATGGG	GGCTGACAGAGGACTTCTCCA
AMPL7155947801	75641275	75641600	Pool 1	CCAGCCAGTTTGTGAATGAGG	GCGGATGTCCACGAAACATAGG
AMPL7157141596	75641540	75641873	Pool 2	CCTGCGCTTTTACACGGC	CCCAATTAGTTTTGAGTTTCCATGC
AMPL7157141571	75641842	75642215	Pool 1	CCAGTGGGCATGGAAACTCAA	CCCATCCTCCTCTTGGCTCT
AMPL7157136034	75642198	75642401	Pool 2	AGCCAAGAGGAGGATGGGTAG	GAAGCCATGTCATGAGATAGTCCC
AMPL7157141573	75642345	75642686	Pool 1	GGCTGAGCGGAATTGTTTCAAG	CCTATCCTATGTGAACATCAGTTCTGTG
AMPL7157141574	75642622	75642981	Pool 2	GTACAGCAGTGAACAAGATGAATGTG	TAAGGCACCAGAGACATCTCCAA
AMPL7157141575	75642924	75643270	Pool 1	TGACCCTTAGGAGACTTGGATACC	GTGATGAGTGGGTCAGAAGACA
AMPL7157141576	75643216	75643491	Pool 2	CCAGGCTACCCTTGTACCTTCA	TGGGTTAGAGACCATATGTGGGT
AMPL7157141577	75643434	75643808	Pool 1	AGGATCTTGAGAAGAATGGACCCT	CATCTCTACCAAAAATTTAAGAAGTAGC CAG
AMPL7157141578	75643733	75644083	Pool 2	CAACCTCCTGATTAACTGGAACCA	GCCTGAAGCAGCAGAATCTCTG
AMPL7157141598	75643903	75644277	Pool 1	CACCAGCCTCAAGTTACTTTAGATCA	TCATTGTGAAAGGACGGCCA
AMPL7157141581	75644256	75644578	Pool 2	CCTGGCCGTCCTTTCACAA	TGCTGACCGGTACAGGATCT
AMPL7157141582	75644524	75644898	Pool 1	GTTCTTCAATGGCATTGGCAACTA	GTAAAGGCAGCTGACCTTCTCT
AMPL7157141583	75644845	75645205	Pool 2	GGATGAACTGCCCAAAGTCTGA	CCCAGCCTGGAAACACTATTGA
AMPL7157141584	75645157	75645528	Pool 1	ACTCAATGGACTAGGCCTCCT	TGGGCAGCTTGGAGGAAAC
AMPL7157134531	75645501	75645659	Pool 2	AAAAACAGTGTTTCCTCCAAGCT	CCCAGTTTGAGGCTAGTCCAG

AMPL7157141586	75645585	75645939	Pool 1	CACACACGCACGTTTATATATATTTTTGGT	TGTGACCTCAGCTGGTTTCTTC
AMPL7157141587	75645880	75646238	Pool 2	TAATCCCACTCCAGGATGGGAA	GTCTCTGCCTGTGTGAACAGTAG
AMPL7157141588	75646185	75646483	Pool 1	ATGGCCGTACCATCTGGTTC	CCCTTAGGAAGCCTCTAGGACA
AMPL7155947798	75646429	75646800	Pool 2	CAGCTTTGCTGATGTGGACTCT	CTTGGATTTCTTTTTGCGGGACTT
AMPL7157141589	75646735	75647070	Pool 1	GATAGGACCCTCCAACTCCAAC	TGGGTTGCAGTCCTCTTAGGAA
AMPL7157136027	75647019	75647250	Pool 2	CTTCCAGGACACGAAGGGCAAA	GCCTTTCTCCCACCTTTGTACAC
AMPL7153478917	75647190	75647464	Pool 1	GCTCTACAGGAGAGGATGGGAT	TCAGATATTGCCTGCTCCCAAAAA
AMPL7157141590	75647406	75647718	Pool 2	TTCTTATTGTCTTGCCCTGCATCT	GATAGAGTCAGAGCCTTGAGGACT

***APOE*:chr19: 45,409,039:45,412,650**					
Amplicon ID	Start	End	Primer Pool	Forward Primer	Reverse Primer
AMPL7152996218	45409014	45409161	Pool 1	GGGAGCCCTATAATTGGACAAGT	AGGGTCCCAGCTCTTTCTAGAG
AMPL7154479522	45409755	45410121	Pool 2	CAACAAGGCTTGGAAGGCTAAC	CAAAACAACTTCTAACTCCTATCTCAAGGA
AMPL7154479521	45410987	45411342	Pool 1	CCTGACCCACCTTGAACTTGTT	ATGGGAAGAGGAAGCTAGAACCA
AMPL7154479523	45411591	45411842	Pool 2	CCTCCTAGCTCCTTCTTCGTCT	CCAGTTCCGATTTGTAGGCCTT
AMPL7154479524	45411792	45412076	Pool 1	CGCTGATGGACGAGACCA	TCTGCAGGTCATCGGCATC
AMPL7154443516	45412028	45412367	Pool 2	CTGCGCAAGCTGCGTAAG	CCTGCAGGCGTATCTGCTG
AMPL7155127581	45412292	45412429	Pool 1	CTGGACGAGGTGAAGGAGCAG	GCGCTGCATGTCTTCCAC
AMPL7155127582	45412385	45412685	Pool 2	CGCCTCAAGAGCTGGTTCG	GGGCTTAGAGGAAATCACAGGG

**APOE PROMOTER:chr19:45,408,011:45,409,011**					
Amplicon ID	Start	End	Primer Pool	Forward Primer	Reverse Primer
AMPL7160266886	45407922	45408265	Pool 1	GGCTGTCCTCCATGCTCAATAC	GCAAATGGGACCTGGGTTTTAATCA
AMPL7160266817	45408209	45408387	Pool 2	TGTGCTCAAGGTCACAACCAA	CCTCAGCAAGAGGGAGACTGT
AMPL7157901648	45408366	45408740	Pool 1	GACAGTCTCCCTCTTGCTGAG	CCTGGATGAATGTAATCTGGAGAGG
AMPL7160266885	45408668	45409040	Pool 2	CCTAGCCCTACTTTCTTTCTGGGAT	CCAGACTTGTCCAATTATAGGGCT
