# Supplementary material for: Investigation of base excision repair gene variants in late-onset Alzheimer’s disease
Source: PLoS One. 2019 Aug 15;14(8):e0221362. doi: 10.1371/journal.pone.0221362 (PMC6695184; doi:10.1371/journal.pone.0221362)
Supplement: S2 Table — (PDF) [file pone.0221362.s004.pdf]

S2 Table. Primers for Sanger sequencing.

Primer ID	Location	Forward primer/ Reverse primer	Annealing Temperature
POLβ	42,220,710	GCCATTCTGAGTGTGTGACTTC/ AAGAGCTGGGACTACACCACCA	62°C
	42,221,461	TGTGATAGAACACTCAGGGGA/ GAATCTTAATTCTAGGCTGGGCA	58°C
	42,203,655	TTGGATTAGTGATACTCAAC/ ACCTGGACTACTCAAAAGG	54°C
UNG	109,535,809 109,535,857	GCGCCTCTGACTCGGTAAAC/ CGTGGTCTTTTCGCGGCA	58.5°C
	109,537,423 109,537,562	TCCCGCGGTGTCAGATGTT/ ACCAGGTTTCATTGGTCTTGAACTC	58.5°C
	109,545,951	GGCACAAAATGGAAAGTAAATGGC/ TCCCAGCCAAGAGACCCTG	58.5°C
	109,546,114	GTACAACTGGTCCCTGGCTTA/ CCACCTCCTAGGCTCAAGTTAC	58.5°C
	109,546,886	CTCTTGGTCCCATGCAGTT/ TGCAAAGAGCACCACCCTC	58.5°C
NEIL1	75,643,714	ATGGCAAACCCACATATGGTC/ GGCAACAAGGAAGACCCCAT	62°C
	75,645,383	GCACTTGTCCCTCTCTGGA/ CTCATGGGCAGCTTGGAGGA	62°C
	75,645,565	ACAGTGTTTCCTCCAAGCTG/ TCCTGGAGTGGGATTAAGAA	58.5°C
	75,645,965	CCTGGACTAGCCTCAAACTG/ CAGAGGGCAGGCTCAGGTT	62°C
APOE	45,410,002	ACAGGCAGGAAGATGAAGT/ CCAGAGAGCGTCAAATCGCT	58.5°C
