# Supplementary material for: Investigation of base excision repair gene variants in late-onset Alzheimer’s disease
Source: PLoS One. 2019 Aug 15;14(8):e0221362. doi: 10.1371/journal.pone.0221362 (PMC6695184; doi:10.1371/journal.pone.0221362)
Supplement: S4 Table — (PDF) [file pone.0221362.s006.pdf]

S4 Table. Minor allele frequencies of the significant variants.

	MA	MAF in our population	ALL	AFR	AMR	EAS	EUR	SAS
**rs1610925**	TA	0.12	0.25	0.42	0.18	0.10	0.22	0.23
**rs2268406**	G	0.14	0.14	0.22	0.12	0.04	0.16	0.15
**rs80001089**	G	0.09	0.03	0.00	0.05	0.00	0.09	0.03
**rs1018782**	G	0.13	0.21	0.44	0.15	0.04	0.16	0.15
**rs1018783**	A	0.12	0.19	0.40	0.15	0.04	0.16	0.15
**rs2430678**	A	0.01	0.05	0.03	0.03	0.06	0.04	0.07
**rs769449**	G	0.10	0.06	0.01	0.08	0.08	0.12	0.06
**rs429358**	C	0.15	0.15	0.27	0.10	0.09	0.16	0.09

MA, minor allele; MAF, minor allele frequency; ALL, all 1000G phase 3 individuals; AFR: African, AMR: American, EAS: East Asian, EUR: European, SAS: South Asian.
