# Supplementary material for: Investigation of base excision repair gene variants in late-onset Alzheimer’s disease
Source: PLoS One. 2019 Aug 15;14(8):e0221362. doi: 10.1371/journal.pone.0221362 (PMC6695184; doi:10.1371/journal.pone.0221362)
Supplement: S5 Table — (PDF) [file pone.0221362.s007.pdf]

S5 Table. Epistatic interaction between statistically significant gene variants in blood samples.

	Variations	LOAD Frequency	Control Frequency	OR (95% CI)	Fisher`s p-value
APOE-APOE	rs429358-rs769449	0.247	0.051	6.12 (2.35-15.91)	0.0001
UNG-UNG	rs80001089-rs1610925	0.212	0.092	2.66 (1.24-5.72)	0.0002
UNG-UNG	rs1610925-rs2268406	0.308	0.163	2.28 (1.23-4.22)	0.0075
UNG-UNG	rs80001089-rs2268406	0.212	0.092	2.66 (1.24-5.72)	0.0091
UNG-UNG	rs80001089-rs1018782	0.202	0.092	2.50 (1.16-5.40)	0.0194
UNG-UNG	rs2268406-rs1018782	0.283	0.163	2.02 (1.09-3.75)	0.0304
UNG-UNG	rs1610925-rs1018782	0.283	0.163	2.02 (1.09-3.75)	0.0304
UNG-UNG	rs2268406-rs1018783	0.247	0.153	1.82 (0.96-3.44)	0.0723
UNG-UNG	rs1018782-rs1018783	0.247	0.153	1.82 (0.96-3.44)	0.0723
UNG-UNG	rs1018783-rs1610925	0.247	0.153	1.82 (0.96-3.44)	0.0723
APOE-UNG	rs769449-rs2268406	0.086	0.031	2.97 (0.85-10.41)	0.0876
APOE-UNG	rs769449-rs1610925	0.091	0.031	3.16 (0.91-11.02)	0.0894
APOE-UNG	rs769449-rs1018782	0.076	0.031	2.60 (0.73-9.20)	0.1948
POLβ – POLβ	rs11993638-rs3136788	0.081	0.041	2.06 (0.67-6.35)	0.2284
POLβ - POLβ	rs3136744-rs3136788	0.081	0.041	2.06 (0.67-6.35)	0.2284
APOE-UNG	rs429358-rs2268406	0.096	0.051	1.97 (0.71-5.46)	0.2578
APOE-NEIL1	rs769449-rs11634109	0.066	0.031	2.22 (0.62-7.99)	0.2795
