# Supplementary material for: Investigation of base excision repair gene variants in late-onset Alzheimer’s disease
Source: PLoS One. 2019 Aug 15;14(8):e0221362. doi: 10.1371/journal.pone.0221362 (PMC6695184; doi:10.1371/journal.pone.0221362)
Supplement: S6 Table — (PDF) [file pone.0221362.s008.pdf]

S6 Table. Allele and genotype frequencies of *UNG*, *POLβ*, *NEIL1* and *APOE* in CE samples of LOAD patients, age-matched cognitively normal and hpC subjects (LOAD=11, hpC=11, Control=10).

LOAD vs Control												
	Allele frequency					Genotype frequency						
	Allele	LOAD	Control	OR (95% CI)	Fisher's p-value	Genotype	LOAD	Control	OR (95% CI)	Fisher's p-value	Pearson $\chi 2$	P value
***UNG*, chr12**												
**rs2569987**	**T**	**0.73**	**1.00**	**-**	**0.0216**	**T/T**	**0.55**	**1.00**			**5.966**	**0.0146**
	**C**	**0.27**	**0.00**			**T/C**	**0.36**	**0.00**	**-**	**0.0867**		
						**C/C**	**0.09**	**0.00**	**-**	**0.4118**		
						**T/C+C/C**	**0.45**	**0.00**	**-**	**0.0351**		
rs2268406	T	0.82	0.95	4.22 (0.43-41.45)	0.3465	T/T	0.64	0.90			2.007	0.1566
	G	0.18	0.05			T/G	0.36	0.10	5.14 (0.46-56.89)	0.3108		
						G/G	0.00	0.00	-	1.0000		
						T/G+G/G	0.36	0.10	5.14 (0.46-56.89)	0.3108		
rs80001089	T	0.91	1.00	-	0.4890	T/T	0.82	1.00			2.010	0.1563
	G	0.09	0.00			T/G	0.18	0.00	-	0.4762		
						G/G	0.00	0.00	-	1.0000		
						T/G+G/G	0.18	0.00	-	0.4762		
***NEIL1*, chr15**												
rs7182283	G	0.45	0.60	1.80 (0.53-6.14)	0.3743	G/G	0.27	0.50			1.147	0.2841
	T	0.55	0.40			G/T	0.36	0.20	3.33 (0.36-30.70)	0.5921		
						T/T	0.36	0.30	2.22 (0.28-17.63)	0.6193		
						G/T+T/T	0.73	0.50	2.67 (0.43-16.39)	0.3870		
***APOE*, chr19**												
rs429358 Cys130Arg	T	0.68	0.80	1.87 (0.45-7.69)	0.4913	T/T	0.45	0.60			0.444	0.5051
	C	0.32	0.20			T/C	0.45	0.40	1.50 (0.26-8.82)	1.0000		
						C/C	0.09	0.00	-	1.0000		
						T/C+C/C	0.55	0.40	1.80 (0.32-10.20)	0.6699		

**hpC vs Control**												
	**Allele frequency**					**Genotype frequency**						
	**Allele**	**hpC**	**Control**	**OR (95% CI)**	**Fisher's p-value**	**Genotype**	**LOAD**	**Control**	**OR (95% CI)**	**Fisher's p-value**	**Pearson χ2**	**P value**
***UNG*, chr12**												
rs2569987	T	0.91	1.00	-	0.4890	T/T	0.82	1.00			2.010	0.1563
	C	0.09	0.00			T/C	0.18	0.00	-	0.4762		
						C/C	0.00	0.00	-	1.0000		
						T/C+C/C	0.18	0.00	-	0.4762		
***POLβ*, chr8**												
rs4526344	G	0.86	1.00	-	0.2334	G/G	0.73	1.00			3.182	0.0745
	C	0.14	0.00			G/C	0.27	0.00	-	0.2143		
						C/C	0.00	0.00	-	1.0000		
						G/C+C/C	0.27	0.00	-	0.2143		
***APOE*, chr19**												
**rs405509**	**T**	**0.73**	**1.00**	**-**	**0.0216**	**T/T**	**0.55**	**1.00**			**5.966**	**0.0146**
	**G**	**0.27**	**0.00**			**T/G**	**0.36**	**0.00**	**-**	**0.0867**		
						**G/G**	**0.09**	**0.00**	**-**	**0.4118**		
						**T/G+G/G**	**0.45**	**0.00**	**-**	**0.0351**		
**LOAD vs hpC+Control**												
	**Allele frequency**					**Genotype frequency**						
	**Allele**	**LOAD**	**Control**	**OR (95% CI)**	**Fisher's p-value**	**Genotype**	**LOAD**	**Control**	**OR (95% CI)**	**Fisher's p-value**	**Pearson χ2**	**P value**
***UNG*, chr12**												
**rs2569987**	**T**	**0.73**	**0.95**	**7.50 (1.37-41.14)**	**0.0162**	**T/T**	**0.55**	**0.90**			**5.453**	**0.0195**
	**C**	**0.27**	**0.05**			**T/C**	**0.36**	**0.10**	**6.33 (0.92-43.62)**	**0.0674**		
						**C/C**	**0.09**	**0.00**	**-**	**0.2692**		
						**T/C+C/C**	**0.45**	**0.10**	**7.92 (1.21-51.84)**	**0.0318**		
rs2268406	T	0.82	0.95	4.44 (0.75-26.52)	0.1697	T/T	0.64	0.90			3.413	0.0647

	G	0.18	0.05			T/G	0.36	0.10	5.43 (0.81-36.51)	0.1476		
						G/G	0.00	0.00	-	1.0000		
						T/G+G/G	0.36	0.10	5.43 (0.81-36.51)	0.1476		
rs80001089	T	0.91	1.00	-	0.1146	T/T	0.82	1.00			4.073	0.0436
	G	0.09	0.00			T/G	0.18	0.00	-	0.1109		
						G/G	0.00	0.00	-	1.0000		
						T/G+G/G	0.18	0.00	-	0.1109		
***NEIL1*, chr15**												
75,641,932	A	0.91	1.00	-	0.1146	A/A	0.82	1.00			4.073	0.0436
	G	0.09	0.00			A/G	0.18	0.00	-	0.1109		
						G/G	0.00	0.00	-	1.0000		
						A/G+G/G	0.18	0.00	-	0.1109		
rs7182283	G	0.45	0.60	1.76 (0.62-4.99)	0.3044	G/G	0.27	0.43			0.748	0.3871
	T	0.55	0.40			G/T	0.36	0.33	1.71 (0.29-10.30)	0.6668		
						T/T	0.36	0.24	2.40 (0.38-15.32)	0.3972		
						G/T+T/T	0.73	0.57	2.00 (0.41-97.74)	0.4647		
***POLβ*, chr8**												
rs3136791	T	0.91	0.98	4.10 (0.35-47.96)	0.2698	T/T	0.82	0.95			1.800	0.1798
	G	0.09	0.02			T/G	0.18	0.05	4.44 (0.36-55.58)	0.2661		
						G/G	0.00	0.00	-	1.0000		
						T/G+G/G	0.18	0.05	4.44 (0.36-55.58)	0.2661		
rs2976238	T	0.68	0.81	1.98 (0.61-6.47)	0.3522	T/T	0.45	0.62			0.794	0.3730
	G	0.32	0.19			T/G	0.45	0.38	1.63 (0.36-7.43)	0.7007		
						G/G	0.09	0.00	-	0.3158		
						T/G+G/G	0.55	0.38	1.95 (4.44-8.55)	0.4651		
***APOE*, chr19**												
rs429358	T	0.68	0.83	2.33 (0.70-7.82)	0.2079	T/T	0.45	0.67			1.347	0.2459
	C	0.32	0.17			T/C	0.45	0.33	2.00 (0.43-9.29)	0.4472		
						C/C	0.09	0.00	-	0.3000		
						T/C+C/C	0.55	0.33	2.40 (0.54-10.69)	0.2826		
