# Supplementary material for: Investigation of base excision repair gene variants in late-onset Alzheimer’s disease
Source: PLoS One. 2019 Aug 15;14(8):e0221362. doi: 10.1371/journal.pone.0221362 (PMC6695184; doi:10.1371/journal.pone.0221362)
Supplement: S7 Table — (PDF) [file pone.0221362.s009.pdf]

S7 Table. Allele and Genotype frequencies of *UNG*, *POLβ*, *NEIL1* and *APOE* in CE and TC of same LOAD patients, age-matched cognitively normal and hpC subjects (LOAD=10, hpC=8, Control=9).

**LOAD vs Control, CE**												
	**Allele frequency**					**Genotype frequency**						
	**Allele**	**LOAD**	**Control**	**OR (95% CI)**	**Fisher's p-value**	**Genotype**	**LOAD**	**Control**	**OR (95% CI)**	**Fisher's p-value**	**Pearson χ2**	**P value**
***UNG*, chr12**												
rs2569987	T	0.80	1.00	-	0.1071	T/T	0.60	1.00			4.560	0.0327
	C	0.20	0.00			T/C	0.40	0.00	-	0.0867		
						C/C	0.00	0.00	-	1.0000		
						T/C+C/C	0.40	0.00	-	0.0867		
rs2268406	T	0.80	0.94	4.25 (0.43-42.19)	0.3436	T/T	0.60	0.89			2.039	0.1533
	G	0.20	0.06			T/G	0.40	0.11	5.33 (0.47-60.80)	0.3034		
						G/G	0.00	0.00	-	1.0000		
						T/G+G/G	0.40	0.11	5.33 (0.47-60.80)	0.3034		
rs80001089	T	0.90	1.00	-	0.4879	T/T	0.80	1.00			2.012	0.1561
	G	0.10	0.00			T/G	0.20	0.00	-	0.4737		
						G/G	0.00	0.00	-	1.0000		
						T/G+G/G	0.20	0.00	-	0.4737		
***NEIL1*, chr15**												
rs7182283	G	0.40	0.67	3.00 (0.80-11.31)	0.1192	G/G	0.20	0.56			2.574	0.1087
	T	0.60	0.33			G/T	0.40	0.22	5.00 (0.47-52.96)	0.2865		
						T/T	0.40	0.22	5.00 (0.47-52.96)	0.1698		
						G/T+T/T	0.80	0.44	5.00 (0.66-38.15)	0.2865		
***APOE*, chr19**												
rs429358 Cys130Arg	T	0.65	0.78	1.88 (0.45-7.97)	0.4848	T/T	0.40	0.56			0.460	0.4977
	C	0.35	0.22			T/C	0.50	0.44	1.56 (0.24-10.03)	1.0000		
						C/C	0.10	0.00	-	1.0000		
						T/C+C/C	0.60	0.44	1.88 (0.30-11.63)	0.6563		

**hpC vs Control, CE**												
	**Allele frequency**					**Genotype frequency**						
	**Allele**	**hpC**	**Control**	**OR (95% CI)**	**Fisher's p-value**	**Genotype**	**LOAD**	**Control**	**OR (95% CI)**	**Fisher's p-value**	**Pearson $\chi 2$**	**P value**
***UNG*, chr12**												
rs2569987	T	0.88	1.00	-	0.2139	T/T	0.75	1.00			2.550	0.1103
	C	0.13	0.00			T/C	0.25	0.00	-	0.2059		
						C/C	0.00	0.00	-	1.0000		
						T/C+C/C	0.25	0.00	-	0.2059		
***POLβ*, chr8**												
rs4526344	G	0.88	1.00	-	0.2139	G/G	0.75	1.00			2.550	0.1103
	C	0.13	0.00			G/C	0.25	0.00	-	0.2059		
						C/C	0.00	0.00	-	1.0000		
						G/C+C/C	0.25	0.00	-	0.2059		
***APOE*, chr19**												
**rs405509**	**T**	**0.63**	**1.00**	**-**	**0.0060**	**T/T**	**0.38**	**1.00**			**7.969**	**0.0048**
	**G**	**0.37**	**0.00**			**T/G**	**0.50**	**0.00**	**-**	**0.0192**		
						**G/G**	**0.13**	**0.00**	**-**	**0.3077**		
						**T/G+G/G**	**0.63**	**0.00**	**-**	**0.0090**		
**LOAD vs hpC+Control, CE**												
	**Allele frequency**					**Genotype frequency**						
	**Allele**	**LOAD**	**Control**	**OR (95% CI)**	**Fisher's p-value**	**Genotype**	**LOAD**	**Control**	**OR (95% CI)**	**Fisher's p-value**	**Pearson $\chi 2$**	**P value**
***UNG*, chr12**												
rs2569987	T	0.80	0.94	4.00 (0.66-24.21)	0.1792	T/T	0.60	0.88			2.904	0.0883
	C	0.20	0.06			T/C	0.40	0.12	5.00 (0.72-34.94)	0.1535		
						C/C	0.00	0.00	-	1.0000		
						T/C+C/C	0.40	0.12	5.00 (0.72-34.94)	0.1535		
rs2268406	T	0.80	0.94	4.00 (0.66-24.21)	0.1792	T/T	0.60	0.88			2.904	0.0883
	G	0.20	0.06			T/G	0.40	0.12	5.00 (0.72-34.94)	0.1535		

						G/G	0.00	0.00	-	1.0000		
						T/G+G/G	0.40	0.12	5.00 (0.72-34.94)	0.1535		
rs80001089	T	0.90	1.00	-	0.1328	T/T	0.80	1.00			3.672	0.0553
	G	0.10	0.00			T/G	0.20	0.00	-	0.1282		
						G/G	0.00	0.00	-	1.0000		
						T/G+G/G	0.20	0.00	-	0.1282		
***NEIL1*, chr15**												
75,641,932	A	0.90	1.00	-	0.1328	A/A	0.80	1.00			3.672	0.0553
	G	0.10	0.00			A/G	0.20	0.00	-	0.1282		
						G/G	0.00	0.00	-	1.0000		
						A/G+G/G	0.20	0.00	-	0.1282		
rs7182283	T	0.40	0.59	2.14 (0.70-6.60)	0.2604	T/T	0.20	0.41			1.271	0.2597
	C	0.60	0.41			T/C	0.40	0.35	2.33 (0.31-17.55)	0.6285		
						C/C	0.40	0.24	3.50 (0.43-28.45)	0.3348		
						T/C+C/C	0.80	0.59	2.80 (0.45-17.38)	0.4059		
***POLβ*, chr8**												
rs3136791	T	0.90	0.97	3.67 (0.31-43.27)	0.5477	T/T	0.80	0.94			1.883	0.1700
	G	0.10	0.03			T/G	0.20	0.06	5.33 (0.41-70.20)	0.2313		
						G/G	0.00	0.00	-	1.0000		
						T/G+G/G	0.20	0.06	5.33 (0.41-70.20)	0.2313		
rs2976238	T	0.65	0.76	1.75 (0.52-5.89)	0.5302	T/T	0.40	0.53			1.724	0.1892
	G	0.35	0.24			T/G	0.50	0.47	2.81 (0.42-18.74)	0.3864		
						G/G	0.10	0.00	-	0.2500		
						T/G+G/G	0.60	0.47	3.38 (0.52-21.73)	0.2337		
***APOE*, chr19**												
rs429358	T	0.65	0.82	2.51 (0.70-8.98)	0.1936	T/T	0.40	0.65			1.556	0.2122
	C	0.35	0.18			T/C	0.50	0.35	2.29 (0.44-11.92)	0.4185		
						C/C	0.10	0.00	-	0.3125		
						T/C+C/C	0.60	0.35	2.75 (0.55-13.75)	0.2566		
**LOAD vs Control, TC**												
	**Allele frequency**					**Genotype frequency**						

	Allele	LOAD	Control	OR (95% CI)	Fisher's p-value	Genotype	LOAD	Control	OR (95% CI)	Fisher's p-value	Pearson χ2	P value
***UNG*, chr12**												
rs80001089	T	0.80	1.00	-	0.1071	T/T	0.60	1.00			4.560	0.0327
	G	0.20	0.00			T/G	0.40	0.00	-	0.0867		
						G/G	0.00	0.00	-	1.0000		
						T/G+G/G	0.00	0.00	-	0.0867		
rs2569987	T	0.80	1.00	-	0.1071	T/T	0.60	1.00			4.560	0.0327
	C	0.20	0.00			T/C	0.40	0.00	-	0.0867		
						C/C	0.00	0.00	-	1.0000		
						T/C+C/C	0.00	0.00	-	0.0867		
rs2268406	T	0.80	0.89	2.00 (0.32-12.51)	0.6630	T/T	0.60	0.78			0.693	0.4052
	G	0.20	0.11			T/G	0.40	0.22	2.33 (0.31-17.55)	0.6285		
						G/G	0.00	0.00	-	1.0000		
						T/G+G/G	0.40	0.22	2.33 (0.31-17.55)	0.6285		
***NEIL1*, chr15**												
rs7182283	G	0.40	0.67	3.00 (0.80-11.31)	0.1192	G/G	0.20	0.56			2.574	0.1087
	T	0.60	0.33			G/T	0.40	0.22	5.00 (0.47-52.96)	0.2861		
						T/T	0.40	0.22	5.00 (0.47-52.96)	0.2861		
						G/T+T/T	0.80	0.44	5.00 (0.66-38.15)	0.1698		
***POLβ*, chr8**												
rs1012381950	T	0.55	0.83	4.09 (0.89-18.72)	0.0860	T/T	0.10	0.67			6.537	0.0106
	C	0.45	0.17			T/C	0.90	0.33	18.00 (1.50-216.63)	0.0198		
						C/C	0.00	0.00	-	1.000		
						T/C+C/C	0.90	0.33	18.00 (1.50-216.63)	0.0198		
***APOE*, chr19**												
rs429358	T	0.65	0.78	1.88 (0.45-7.97)	0.4848	T/T	0.40	0.56			0.460	0.4977
	C	0.35	0.22			T/C	0.50	0.44	1.56 (0.24-10.03)	1.0000		
						C/C	0.10	0.00	-	1.0000		

						T/C+C/C	0.60	0.44	1.88 (0.30-11.63)	0.6563		
**hpC vs Control, TC**												
	**Allele frequency**					**Genotype frequency**						
	**Allele**	**hpC**	**Control**	**OR (95% CI)**	**Fisher's p-value**	**Genotype**	**LOAD**	**Control**	**OR (95% CI)**	**Fisher's p-value**	**Pearson χ2**	**P value**
**UNG, chr12**												
rs2569987	T	0.88	1.00	-	0.2139	T/T	0.75	1.00			2.550	0.1103
	C	0.13	0.00			T/C	0.25	0.00	-	0.2059		
						C/C	0.00	0.00	-	1.0000		
						T/C+C/C	0.25	0.00	-	0.2059		
***POLβ*, chr8**												
rs1012381950	T	0.63	0.83	3.00 (0.61-14.86)	0.2497	T/T	0.25	0.67			2.951	0.0858
	C	0.38	0.17			T/C	0.75	0.33	6.00 (0.72-49.84)	0.1534		
						C/C	0.00	0.00	-	1.0000		
						T/C+C/C	0.75	0.33	6.00 (0.72-49.84)	0.1534		
***APOE*, chr19**												
rs429358	T	0.94	0.83	0.33 (0.03-3.58)	0.6041	T/T	0.88	0.67			1.022	0.3121
	C	0.06	0.17			T/C	0.13	0.33	0.29 (0.02-3.52)	0.5765		
						C/C	0.00	0.00	-	1.0000		
						T/C+C/C	0.13	0.33	0.29 (0.02-3.52)	0.5765		
**LOAD vs hpC+Control, TC**												
	**Allele frequency**					**Genotype frequency**						
	**Allele**	**LOAD**	**Control**	**OR (95% CI)**	**Fisher's p-value**	**Genotype**	**LOAD**	**Control**	**OR (95% CI)**	**Fisher's p-value**	**Pearson χ2**	**P value**
***UNG*, chr12**												
**rs80001089**	**T**	**0.80**	**1.00**	**-**	**0.0153**	**T/T**	**0.60**	**1.00**			**7.983**	**0.0047**
	**G**	**0.20**	**0.00**			**T/G**	**0.40**	**0.00**	**-**	**0.0120**		
						**G/G**	**0.00**	**0.00**	**-**	**1.0000**		
						**T/G+G/G**	**0.40**	**0.00**	**-**	**0.0120**		
rs2569987	T	0.80	0.94	4.00 (0.66-24.21)	0.1792	T/T	0.60	0.88			2.904	0.0883
	C	0.20	0.06			T/C	0.40	0.12	5.00 (0.72-34.92)	0.1535		
						C/C	0.00	0.00	-	1.0000		
						T/C+C/C	0.40	0.12	5.00 (0.72-34.92)	0.1535		
rs1610925	-	0.70	0.85	2.49 (0.65-9.56)	0.2937	-/-	0.40	0.71			2.440	0.1183

	TA	0.30	0.15			-/TA	0.60	0.29	3.60 (0.70-18.56)	0.2238		
						TA/TA	0.00	0.00	-	1.0000		
						-/TA +TA/TA	0.60	0.29	3.60 (0.70-18.56)	0.2238		
rs2268406	T	0.80	0.88	1.88 (0.41-8.51)	0.4495	T/T	0.60	0.76			0.819	0.3654
	G	0.20	0.12			T/G	0.40	0.24	2.17 (0.40-11.74)	0.4147		
						G/G	0.00	0.00	-	1.0000		
						T/G+G/G	0.40	0.24	2.17 (0.40-11.74)	0.4147		
***NEIL1*, chr15**												
rs7182283	G	0.40	0.65	2.75 (0.88-8.58)	0.0955	G/G	0.20	0.47			1.977	0.1597
	T	0.60	0.35			G/T	0.40	0.35	2.67 (0.36-19.71)	0.6285		
						T/T	0.40	0.18	5.33 (0.62-45.99)	0.1618		
						G/T+T/T	0.80	0.53	3.56 (0.58-21.92)	0.2305		
***POLβ*, chr8**												
rs1012381950	T	0.55	0.74	2.27 (0.71-7.28)	0.2330	T/T	0.1	0.5			3.891	0.0485
	C	0.45	0.26			T/C	0.9	0.5	8.00 (0.82-77.82)	0.0912		
						C/C	0.0	0.0	-	1.0000		
						T/C+C/C	0.9	0.5	8.00 (0.82-77.82)	0.0912		
***APOE*, chr19**												
rs429358	T	0.65	0.79	2.08 (0.60-7.17)	0.3368	T/T	0.40	0.59			0.894	0.3445
	C	0.35	0.21			T/C	0.50	0.41	1.79 (0.35-9.13)	0.6828		
						C/C	0.10	0.00	-	0.3333		
						T/C+C/C	0.60	0.41	2.14 (0.44-10.53)	0.4401		
