# Supplementary material for: Investigation of base excision repair gene variants in late-onset Alzheimer’s disease
Source: PLoS One. 2019 Aug 15;14(8):e0221362. doi: 10.1371/journal.pone.0221362 (PMC6695184; doi:10.1371/journal.pone.0221362)
Supplement: S8 Table — (PDF) [file pone.0221362.s010.pdf]

S8 Table. Allele and genotype frequencies of *APOE* ε2, ε3 and ε4 in TC and CE samples of LOAD patients age-matched cognitively normal and hpC subjects (LOAD=10, hpC=8, Control=9).

rs429358-rs7412					
**LOAD vs Control, CE**					
		LOAD	Control	OR (95% CI)	Fisher's p-value
Allele					
ε2	T-T	0.00	0.00	-	1.0000
ε3	T-C	0.65	0.78	(REF)	(REF)
ε4	C-C	0.35	0.22	1.88 (0.45-7.97)	0.4848
Genotype					
ε3/ε4	TT-TT	0.50	0.44	1.56 (0.24-10.03)	1.0000
ε4/ε4	TT-TC	0.10	0.00	-	1.0000
ε2/ε2	TC-TC	0.00	0.00	-	1.0000
ε2/ε3	TT-CC	0.00	0.00	-	1.0000
ε2/ε4	TC-CC	0.00	0.00	-	1.0000
ε3/ε3	CC-CC	0.40	0.56	(REF)	(REF)
**hpC vs Control, CE**					
		hpC	Control	OR (95% CI)	Fisher's p-value
Allele					
ε2	T-T	0.00	0.00	-	1.0000
ε3	T-C	0.88	0.78	(REF)	(REF)
ε4	C-C	0.13	0.22	0.50 (0.08-3.19)	0.6602
Genotype					
ε3/ε4	TT-TT	0.25	0.44	0.42 (0.05-3.31)	0.6199
ε4/ε4	TT-TC	0.00	0.00	-	1.0000
ε2/ε2	TC-TC	0.00	0.00	-	1.0000
ε2/ε3	TT-CC	0.00	0.00	-	1.0000
ε2/ε4	TC-CC	0.00	0.00	-	1.0000
ε3/ε3	CC-CC	0.75	0.56	(REF)	(REF)
**LOAD vs hpC+Control, CE**					
		LOAD	Control	OR (95% CI)	Fisher's p-value
**Allele**					
ε2	T-T	0.00	0.00		
ε3	T-C	0.65	0.82	(REF)	(REF)
ε4	C-C	0.35	0.18	2.51 (0.70-8.98)	0.1936
**Genotype**					
ε3/ε4	TT-TT	0.50	0.35	2.29 (0.44-11.92)	0.4185
ε4/ε4	TT-TC	0.10	0.00	-	0.3125
ε2/ε2	TC-TC	0.00	0.00	-	1.0000
ε2/ε3	TT-CC	0.00	0.00	-	1.0000
ε2/ε4	TC-CC	0.00	0.00	-	1.0000
ε3/ε3	CC-CC	0.40	0.65	(REF)	(REF)

LOAD vs Control, TC					
		LOAD	Control	OR (95% CI)	Fisher's p-value
**Allele**					
ε2	T-T	0.00	0.00	-	1.0000
ε3	T-C	0.65	0.78	(REF)	(REF)
ε4	C-C	0.35	0.22	1.88 (0.45-7.97)	0.4848
**Genotype**					
ε3/ε4	TT-TT	0.50	0.44	1.56 (0.24-10.03)	1.0000
ε4/ε4	TT-TC	0.10	0.00	-	1.0000
ε2/ε2	TC-TC	0.00	0.00	-	1.0000
ε2/ε3	TT-CC	0.00	0.00	-	1.0000
ε2/ε4	TC-CC	0.00	0.00	-	1.0000
ε3/ε3	CC-CC	0.40	0.56	(REF)	(REF)
**hpC vs Control, TC**					
		hpC	Control	OR (95% CI)	Fisher's p-value
**Allele**					
ε2	T-T	0.00	0.00	-	1.0000
ε3	T-C	0.81	0.78	(REF)	(REF)
ε4	C-C	0.19	0.22	0.81 (0.15-4.32)	1.0000
**Genotype**					
ε3/ε4	TT-TT	0.38	0.44	0.75 (0.11-5.24)	1.0000
ε4/ε4	TT-TC	0.00	0.00	-	1.0000
ε2/ε2	TC-TC	0.00	0.00	-	1.0000
ε2/ε3	TT-CC	0.00	0.00	-	1.0000
ε2/ε4	TC-CC	0.00	0.00	-	1.0000
ε3/ε3	CC-CC	0.62	0.56	(REF)	(REF)
**LOAD vs hpC+Control, TC**					
		LOAD	Control	OR (95% CI)	Fisher's p-value
**Allele**					
ε2	T-T	0.00	0.00	-	1.0000
ε3	T-C	0.65	0.79	(REF)	(REF)
ε4	C-C	0.35	0.21	1.12 (0.46-2.75)	0.3368
**Genotype**					
ε3/ε4	TT-TT	0.50	0.42	1.79 (0.35-9.13)	0.6828
ε4/ε4	TT-TC	0.10	0.00	-	0.3333
ε2/ε2	TC-TC	0.00	0.00	-	1.0000
ε2/ε3	TT-CC	0.00	0.00	-	1.0000
ε2/ε4	TC-CC	0.00	0.00	-	1.0000
ε3/ε3	CC-CC	0.40	0.58	(REF)	(REF)
