# Supplementary material for: Investigation of base excision repair gene variants in late-onset Alzheimer’s disease
Source: PLoS One. 2019 Aug 15;14(8):e0221362. doi: 10.1371/journal.pone.0221362 (PMC6695184; doi:10.1371/journal.pone.0221362)
Supplement: S9 Table — (PDF) [file pone.0221362.s011.pdf]

S9 Table. Effect of the interaction of variants with *APOE* ε4 in CE and TC samples of LOAD patients, age-matched cognitively normal and hpC subjects (LOAD=10, hpC=8, Control=9).

**LOAD vs Control, CE**					
		**LOAD**	**Control**	**OR (95% CI)**	**Fisher's p-value**
APOE ε4 (+)	rs2569987 (+)	0.20	0.00	-	0.1667
	rs2569987 (-)	0.20	0.56		
APOE ε4 (-)	rs2569987 (+)	0.20	0.00	-	0.4667
	rs2569987 (-)	0.40	0.44		
rs2569987 (+)	APOE ε4 (+)	0.20	0.00	-	-
	APOE ε4 (-)	0.20	0.00		
rs2569987 (-)	APOE ε4 (+)	0.20	0.56	0.40 (0.05-3.42)	0.6084
	APOE ε4 (-)	0.40	0.44		
**hpC vs Control, CE**					
		**hpC**	**Control**	**OR (95% CI)**	**Fisher's p-value**
APOE ε4 (+)	rs405509 (+)	0.125	0.00	-	0.3750
	rs405509 (-)	0.250	0.56		
APOE ε4 (-)	rs405509 (+)	0.500	0.00	-	0.0476
	rs405509 (-)	0.125	0.44		
rs405509 (+)	APOE ε4 (+)	0.125	0.00	-	-
	APOE ε4 (-)	0.500	0.00		
rs405509 (-)	APOE ε4 (+)	0.250	0.56	1.60 (0.10-24.70)	1.0000
	APOE ε4 (-)	0.125	0.44		
**LOAD vs hpC+Control, CE**					
		**LOAD**	**Control**	**OR (95% CI)**	**Fisher's p-value**
APOE ε4 (+)	rs2569987 (+)	0.20	0.06	11.00 (0.65-187.18)	0.1357
	rs2569987 (-)	0.20	0.65		
APOE ε4 (-)	rs2569987 (+)	0.20	0.00	-	0.4545
	rs2569987 (-)	0.40	0.29		
rs2569987 (+)	APOE ε4 (+)	0.20	0.06	-	1.0000
	APOE ε4 (-)	0.20	0.00		
rs2569987 (-)	APOE ε4 (+)	0.20	0.65	0.23 (0.03-1.68)	0.1778
	APOE ε4 (-)	0.40	0.29		
**LOAD vs hpC+Control, TC**					
		**LOAD**	**Control**	**OR (95% CI)**	**Fisher's p-value**
APOE ε4 (+)	rs80001089 (+)	0.10	0.00	-	0.1667
	rs80001089 (-)	0.10	0.59		
APOE ε4 (-)	rs80001089 (+)	0.30	0.00	-	0.2000
	rs80001089 (-)	0.50	0.41		
rs80001089 (+)	APOE ε4 (+)	0.10	0.00	-	-
	APOE ε4 (-)	0.30	0.00		
rs80001089 (-)	APOE ε4 (+)	0.10	0.59	0.14 (0.01-1.47)	0.1550
	APOE ε4 (-)	0.50	0.41		
