# Supplementary material for: Investigation of base excision repair gene variants in late-onset Alzheimer’s disease
Source: PLoS One. 2019 Aug 15;14(8):e0221362. doi: 10.1371/journal.pone.0221362 (PMC6695184; doi:10.1371/journal.pone.0221362)
Supplement: S10 Table — (PDF) [file pone.0221362.s012.pdf]

S10 Table. Common variations in Turkish population

					MAF					
**Gene**	**Variation**	**MA**	**Genomic location**	**MAF in our population**	**ALL**	**AFR**	**AMR**	**EAS**	**EUR**	**SAS**
NEIL1	rs7182283	G	intronic	0.47	0.53	0.74	0.37	0.48	0.52	0.44
APOE	rs405509	G	upstream	0.44	0.53	0.76	0.5	0.33	0.52	0.45
UNG	rs2541886	T	intronic	0.44	0.25	0.04	0.29	0.19	0.50	0.30
NEIL1	rs5745920	T	downstream	0.39	0.39	0.58	0.24	0.34	0.28	0.39
UNG	rs246079	G	intronic	0.36	0.62	0.75	0.66	0.71	0.41	0.53
		A		0.64	0.38	0.25	0.34	0.29	0.59	0.47
UNG	rs3219211	C	intronic	0.18	0.23	0.20	0.15	0.30	0.17	0.30
UNG	rs3219243	C	intronic	0.18	0.23	0.22	0.15	0.3	0.17	0.27
APOE	rs449647	T	upstream	0.18	0.20	0.33	0.26	0.30	0.17	0.19
POLβ	rs35172933	AGG	intronic	0.17	0.35	0.84	0.14	0.17	0.12	0.28
POLβ	rs2976239	C	intronic	0.17	0.13	0.39	0.05	0.01	0.04	0.02
POLβ	rs2976238	T	intronic	0.16	0.30	0.72	0.10	0.17	0.08	0.25
POLβ	rs2979896	C	intronic	0.15	0.31	0.74	0.10	0.17	0.09	0.25
APOE	rs429358	C	exonic	0.15	0.15	0.27	0.10	0.09	0.16	0.09
POLβ	rs6474390	T	intronic	0.15	0.31	0.74	0.10	0.17	0.09	0.25
UNG	rs2268406	G	intronic	0.14	0.14	0.22	0.12	0.04	0.16	0.15
POLβ	rs2979895	G	intronic	0.14	0.30	0.72	0.10	0.17	0.09	0.25
POLβ	rs2953983	G	intronic	0.14	0.30	0.72	0.10	0.17	0.09	0.25
POLβ	rs3136717	C	intronic	0.14	0.34	0.77	0.14	0.17	0.12	0.28
POLβ	rs3136718	A	intronic	0.13	0.31	0.74	0.10	0.17	0.09	0.25
UNG	rs1018782	G	intronic	0.13	0.21	0.44	0.15	0.04	0.16	0.15
POLβ	rs2953993	T	intronic	0.13	0.30	0.72	0.10	0.17	0.09	0.25
UNG	rs3219235	T	intronic	0.13	0.23	0.20	0.15	0.30	0.17	0.30
UNG	rs1610925	TA	intronic	0.12	0.25	0.42	0.18	0.10	0.22	0.23
POLβ	rs3136790	C	intronic	0.12	0.34	0.79	0.14	0.17	0.12	0.28
POLβ	rs2272615	G	intronic	0.12	0.33	0.77	0.13	0.17	0.12	0.28
POLβ	rs3136781	C	intronic	0.12	0.34	0.79	0.14	0.17	0.13	0.28
POLβ	rs2976244	T	intronic	0.12	0.31	0.74	0.10	0.17	0.09	0.25
UNG	rs1018783	A	intronic	0.12	0.19	0.40	0.15	0.04	0.16	0.15
POLβ	rs3136794	G	intronic	0.11	0.33	0.76	0.14	0.17	0.12	0.28
NEIL1	rs11634109	C	intronic	0.10	0.10	0.07	0.11	0.03	0.24	0.05
APOE	rs769449	A	intronic	0.10	0.06	0.01	0.08	0.08	0.12	0.06
POLβ	rs3136738	T	intronic	0.10	0.31	0.74	0.10	0.17	0.09	0.25
POLβ	rs2953994	G	intronic	0.10	0.31	0.74	0.10	0.17	0.09	0.25
UNG	rs80001089	G	intronic	0.09	0.03	0.00	0.05	0.00	0.09	0.03
POLβ	rs3136811	G	intronic	0.09	0.31	0.74	0.10	0.17	0.09	0.25
POLβ	rs3136722	T	intronic	0.09	0.13	0.40	0.05	0.01	0.04	0.02
UNG	109,541,016	C	intronic	0.08	-	-	-	-	-	-
NEIL1	rs5745925	-	exonic	0.08	0.03	0.01	0.05	0.00	0.08	0.02

POLβ	rs35609234	-	intronic	0.08	0.34	0.77	0.14	0.17	0.12	0.28
POLβ	rs11990332	G	intronic	0.08	0.34	0.77	0.14	0.17	0.12	0.28
POLβ	rs59423074	C	intronic	0.08	0.31	0.74	0.10	0.17	0.09	0.25
POLβ	rs3136780	A	intronic	0.08	0.30	0.72	0.10	0.17	0.09	0.25
POLβ	rs3136793	G	intronic	0.08	0.30	0.72	0.10	0.17	0.09	0.25
POLβ	rs2976240	G	intronic	0.08	0.06	0.15	0.04	0.01	0.04	0.02
UNG	rs2569987	C	intronic	0.07	0.06	0.03	0.10	0.00	0.16	0.04
POLβ	rs3136806	G	intronic	0.07	0.22	0.42	0.17	0.14	0.10	0.22
POLβ	rs141279009	A	intronic	0.07	0.01	0.00	0.01	0.00	0.01	0.03
UNG	rs246078	A	intronic	0.06	0.00	0.00	0.00	0.00	0.02	0.00
APOE	rs769446	C	upstream	0.06	0.06	0.03	0.04	0.11	0.08	0.07
NEIL1	rs5745916	A	intronic	0.05	0.01	0.00	0.01	0.00	0.03	0.01
POLβ	rs3136748	T	intronic	0.05	0.18	0.33	0.05	0.16	0.04	0.23
POLβ	rs2307158	T	upstream	0.05	0.22	0.48	0.05	0.16	0.05	0.23

MA, minor allele; MAF, minor allele frequency; ALL, all 1000G phase 3 individuals; AFR: African, AMR: American, EAS: East Asian, EUR: European, SAS: South Asian.
