# Supplementary material for: Investigation of base excision repair gene variants in late-onset Alzheimer’s disease
Source: PLoS One. 2019 Aug 15;14(8):e0221362. doi: 10.1371/journal.pone.0221362 (PMC6695184; doi:10.1371/journal.pone.0221362)
Supplement: S11 Table — (PDF) [file pone.0221362.s013.pdf]

S11 Table – List of abbreviations

Abbreviation	Complete Name
5-OHU	5-Hydroxyuracil
8-OHGua	8-Hydroxyguanine
AD	Alzheimer's disease
APE1	AP endonuclease 1
APOE	Apolipoprotein E
Arg	Arginine
Aβ	Amyloid-β
BER	Base excision repair
CDR	Clinical dementia rating scale
CE	Cerebellum
CI	95% Confidence interval
CTRL	Age-matched cognitively normal control
Cys	Cysteine
DSM-IV	Diagnostic and statistical manual of mental disorders, 4th ed
dUTP	Deoxyuridine triphosphate
FapyAde	4,6-Diamino-5-formamidopyrimidine
FapyGua	2,6-Diamino-4-hydroxy-5-formamidopyrimidine
GAB2	GRB2 Associated binding protein 2
hpC	High-pathology control
HWE	Hardy-weinberg equilibrium
IGV	Integrative genomics viewer
INDEL	Insertion/deletion
ISP	Ion sphere particle
LD	Linkage disequilibrium
LOAD	Late-onset Alzheimer's disease
MAF	Minor allele frequency
MCI	Mild-cognitive impairment
MMSE	Mini-mental state examination
NEIL1	Endonuclease VIII-like DNA glycosylase 1
NGS	Next generation sequencing
NINCDS-ADRDA	Neurological and communicative disorders and stroke-Alzheimer's disease and related disorders association
OGG1	8-Oxoguanine DNA glycosylase
OR	Odds ratio
PARP1	Poly-ADP-ribose polymerase 1
PGM	Ion torrent personal genome machine
POLβ	Polymerase β

PSEN1	Presenilin-1
Q score	PHRED quality score
ROS	Reactive oxygen species
SD	Standard deviation
SNP	Single nucleotide polymorphism
TC	Temporal cortex
UNG	Uracil DNA glycosylase
UNG1	Mitochondrial uracil DNA glycosylase
UNG2	Nuclear uracil DNA glycosylase
VCF	Variant call format
XRCC1	X-Ray repair cross complementing 1
ε2	ε2 allele of the apolipoprotein E gene
ε3	ε3 allele of the apolipoprotein E gene
ε4	ε4 allele of the apolipoprotein E gene
