# Supplementary material for: Investigation of base excision repair gene variants in late-onset Alzheimer’s disease
Source: PLoS One. 2019 Aug 15;14(8):e0221362. doi: 10.1371/journal.pone.0221362 (PMC6695184; doi:10.1371/journal.pone.0221362)
Supplement: S1 Text — (PDF) [file pone.0221362.s014.pdf]

Materials and Methods

RNA isolation and cDNA synthesis

Fresh peripheral blood samples were collected from 6 LOAD patients who carry statistically significant *UNG* variants (two of them have rs1610925, rs2268406, rs1018782 and rs101878, and four of them have rs1610925, rs2268406, and rs80001089) and 4 age-matched cognitively normal controls who do not have *UNG* variants. Blood samples (10 ml) were applied on top of Histopaque1119 solution (Sigma–Aldrich, USA) and leukocytes were isolated by centrifugation according to the manufacturer's procedure. Total RNA was isolated using RNeasy Mini Kit (Qiagen, Germany) according to the manufacturer`s protocol. RNA quality was evaluated using NanoDrop 2000c Spectrophotometer (Thermo Fisher Scientific, USA). cDNAs were synthesized using iScript™cDNA Synthesis Kit (BioRad, USA) according to the manufacturer's protocol.

Reverse transcription (RT-PCR)

UNG gene expression analysis was performed using PowerUp™ SYBR™ Green Master Mix (Applied Biosystems, USA) and LightCycler® Nano (Roche, Switzerland) instrument according to the manufacturer's protocol. All experiments were performed in triplicate. Following primers were used for *UNG* gene: forward primer: 5'-ACTGTTTATCCACCCCCACAC-3' and reverse primer: 5'-ATGTTCTCCAAACTGGGCGGA-3'. *β-Actin* was used as a housekeeping gene:; forward primer: 5'-CACCAACTGGGACGACAT-3' and reverse primer: 5'-ACAGCCTGGATAGGCAACG-3'. The relative amount of *UNG* mRNA was calculated with normalization of Ct values to the mean Ct value for the *β-actin* reference gene. Data were analyzed using the standard ΔCT and $2^{-\Delta CT}$ method. Student's t-test (Mann-Whitney test) was

used to determine the significance of gene expression differences between case and controls using GraphPad Prism 5.03. P value lower than 0.05 was considered as significant.
